# Mean field analysis of algorithms for scale-free networks in molecular biology

**DOI:** 10.1371/journal.pone.0189866

**Published:** 2017-12-22

**Authors:** S. Konini, E. J. Janse van Rensburg

**Affiliations:** Mathematics & Statistics, York University, Toronto, Ontario, M3J 1P3, Canada; Universitat Rovira i Virgili, SPAIN

## Abstract

The sampling of scale-free networks in Molecular Biology is usually achieved by growing networks from a seed using recursive algorithms with elementary moves which include the addition and deletion of nodes and bonds. These algorithms include the Barabási-Albert algorithm. Later algorithms, such as the Duplication-Divergence algorithm, the Solé algorithm and the iSite algorithm, were inspired by biological processes underlying the evolution of protein networks, and the networks they produce differ essentially from networks grown by the Barabási-Albert algorithm. In this paper the mean field analysis of these algorithms is reconsidered, and extended to variant and modified implementations of the algorithms. The degree sequences of scale-free networks decay according to a powerlaw distribution, namely *P*(*k*) ∼ *k*^−*γ*^, where *γ* is a scaling exponent. We derive mean field expressions for *γ*, and test these by numerical simulations. Generally, good agreement is obtained. We also found that some algorithms do not produce scale-free networks (for example some variant Barabási-Albert and Solé networks).

## Introduction

Many systems in nature and society are described by means of complex networks [1]. Some of these systems include the cell [2], chemical reactions [3], the world wide web [4], social interactions [5], etc. It is generally found that many system, though different in nature, produce networks which are scale-free and exhibit similar properties [6, 7].

The main property of scale-free networks is that their degree distribution decays as a power law [6, 8]—this shows that there is no characteristic scale for the degrees, which is why the networks are called scale-free. The average degree of a scale-free network offers little insight into the real topology of the network [7] since most nodes have degrees which are far away from the average degree of the network. Nodes of high degree are called *hubs* and though small in number for realistic networks, they are over-represented compared to the number of hubs in random networks. These hubs play an important role in dynamical processes which occur in scale-free networks.

Scale-free networks also exhibit an unexpected degree of robustness—this is the property that such networks maintain their dynamic properties even when many nodes and bonds fail to transmit signals (suffer high failure rates) [1]. However, these networks remain vulnerable to failure of hub nodes, since these nodes play a significant role in maintaining the network’s connectivity.

In this paper the mean field approach to the analysis of algorithms for sampling scale-free networks inspired by processes in molecular biology is presented. In addition, numerical testing and, in some cases, verification, of the mean field approach will be examined. The focus will be on four widely used and discussed algorithms in the literature, nameley, the Barabási-Albert algorithm [8, 9], the Duplication-Divergence algorithm [10, 11], the Solé algorithm [12] and the iSite algorithm [13, 14].

The Duplication-Divergence, Solé and iSite algorithms are inspired by modelling networks in biological models of protein-protein interaction evolution, and all these algorithms are based in one way or another on two ideas: growth by preferential attachment [15], and growth and changes (*mutations*) in networks induced by the duplication, deletion or replacement of nodes or bonds (these are elementary moves which *mutate* the network by adding, deleting or moving some of its bonds or nodes).

Growth by preferential attachment is implemented by adding bonds preferentially to nodes of high degree. This increases the probability that a node will grow to be a hub in the network, and the resulting network has an increased probability that it will contain hubs [8]. The Barabási-Albert algorithm uses preferential attachment to grow scale-free networks by attaching bonds to nodes with a probability which is proportional to the degrees of nodes [6]. A mean field analysis of the Barabási-Albert algorithm was done in reference [9].

The Duplication-Divergence algorithm [10, 11] generates scale-free networks by implementing elementary moves which mutate and grow the network. These are *duplication* (the duplication of existing nodes and bonds) and *divergence* (local changes made to existing bonds and nodes) elementary moves. These moves model processes which are thought to underlie the evolutionary mechanisms by which protein interaction networks evolve [10, 11, 16]: The *duplication* of genes is a mechanism which generates genes coding for new proteins during evolution and the *divergence* step is a model for the mutation of duplicated genes. After a duplication of a gene, two genes (one the *progenitor* gene, the other the *progeny* gene) coding for the same protein are obtained, and these mutate over time to drift away from one another in gene space, giving rise to modified proteins when translated by cellular machinery [16]. Biologically, the duplication step may result in a new protein interaction between two mutating copies of the same gene (this is called heteromerization), and the divergence step is a model of subfunctionalization (a process where interactions between proteins are lost).

Closely related to the Duplication-Divergence algorithm is the Solé algorithm [12, 16]. This algorithm grows networks by duplication of nodes, and mutates the network by rewiring it (this algorithm does not implement the heteromerization of the duplicated genes) [4]. It then implements a process of deleting some bonds on the duplicated nodes (modelling evolutionary changes due to subfunctionalization).

The iSite algorithm [13, 14] is a refinement of the Duplication-Divergence and Solé algorithms. This algorithm introduces more complex nodes which each contains *interaction sites* as models of protein and protein complexes with localized interaction sites where the interactions with other proteins take place. These localized interaction sites are *iSites*. Such iSites may be involved in many interactions, but each interaction is related to only two iSites, one on each of the proteins involved. That is, iSites are models of the concept of domains on protein surfaces where the actual interactions take place between two proteins. The implementation of the algorithm on nodes containing iSites proceeds by duplication of nodes, and the mutation of iSites through subfunctionalization and heteromerization (namely, the subfunctionalization of iSites leading to loss of protein interactions, and heteromerization where new interactions are introduced between existing iSites). In this model the subfunctionalization is of iSites, leading to the loss of all bonds incident with the iSite (contrary to the situation in other algorithms, for example the Duplication-Divergence algorithm, where subfunctionalization leads to the loss of bonds, rather than nodes).

This paper is organised as follows. We first consider the general properties of scale-free networks, including their scaling and connectivity properties. These ideas are then applied to the analysis of particular algorithms. The Barabási-Albert model is considered first together with a new modified version of the algorithm, and a new variant of the algorithm. Mean field theory for the modified and variant algorithms is developed, giving mean field values for the scaling exponent *γ*. These results are compared to numerical results obtained by generating networks using implementations of the algorithms.

The Duplication-Divergence algorithm and networks generated by it are considered next. The algorithm is also newly modified, and mean field theory is developed to find mean field values for the scaling exponent. The mean field predictions are then compared to numerical results generated by implementing the algorithm and sampling networks.

A similar approach is followed for the Solé algorithm. However, in this model the degree distribution is not integrable, and our results indicate that the networks generated by this algorithm are not scale-free. Instead, the degree distribution must be modified. This gives a testable scaling hypothesis for Solé networks, which is tested numerically by generating networks and examining their scaling, as well as by computing the connectivity of Solé networks and comparing it to the mean field predictions. This shows that the size of Solé networks of order *n* is *O*(*n*^2^), while the connectivity is *O*(*n*)—this implies that Solé networks are rich in bonds (and are dense networks).

Finally, the iSite algorithm is presented and examined developing a mean field approach to determine its scaling properties. The algorithm is also modified in a new way, and the resulting mean field results are tested numerically.

The paper is completed in the conclusion section, where our main results are briefly considered and reviewed.

## Scale-free networks

Scale-free networks of order *n* are characterised by degree sequences {*d*_*k*_} which follow a power law distribution (where *d*_*k*_ is the number of nodes of degree *k* and $\frac{1}{n}d_{k}$ is the fraction of nodes of degree *k*).

If 〈*d*_*k*_〉 is the average degree distribution, then $\frac{1}{n}\langle d_{k}\rangle$ is proportional to the probability *P*(*k*) that a node has degree *k*. In scale-free networks, the probability *P*(*k*) decays like a powerlaw with exponent *γ*:

$$\begin{array}{c} P\left(k\right)≃C_{o}^{-1}\;k^{-\gamma }. \end{array}$$ (1)

Here, *γ* is the *scale-free network exponent*. The constant *C*_*o*_ is a normalisation constant given by

$$\begin{array}{c} C_{o}=\sum_{k=1}^{n}k^{-\gamma }. \end{array}$$ (2)

As *n* → ∞, it is necessary that *γ* > 1 for *P*(*k*) to be summable (and *C*_*o*_ < ∞). In this case *C*_*o*_ converges to a constant as *n* → ∞. Thus, if *γ* > 1 then the network is said to be integrable with scaling exponent *γ* (in this event Eq (1) is the scaling of the limiting degree distribution with *C*_*o*_ > 0 finite and *P*(*k*)→0 as *k* → ∞).

The case that *γ* = 1 gives rise to a logarithmic correction. Since $\sum_{k=1}^{n}k^{-1}∼\log n$, this gives the distribution

$$\begin{array}{c} P\left(k\right)∼\frac{1}{\log n}\;k^{-1} \end{array}$$ (3)

for networks of (large) order *n*. This network is said to be not integrable, but for asymptotic values and fixed values of *n* the decay of *P*(*k*) will appear to be proportional to *k*^−1^.

Since *P*(*k*) is the probability that a node in a network has degree *k*, the average degree sequence {〈*d*_*k*_〉_*n*_} over randomly generated networks of order *n* is given approximately by 〈*d*_*k*_〉 ∼ *nP*(*k*), for *n* large. It is not known that the degree sequence is self-averaging (that is, that the degree sequence {*d*_*k*_} has distribution *d*_*k*_ ∼ *nP*(*k*) as *n* → ∞ for a single randomly generated scale-free network).

This powerlaw decay of degree sequences shows that nodes of large degree (that is, for large *k*) are more common in scale-free networks (compared to randomly generated networks, where they are exponentially rare). These nodes of large degree are called *hubs*. A precise definition of a hub in a network is somewhat arbitrary, but for the purpose of this paper, a “hub” in a network of order *n* is defined as a node of degree exceeding $\left\lfloor \sqrt{n}\right\rfloor$.

The exponent *γ* can be estimated from numerical data by computing the average degree sequence {〈*d*_*k*_〉} and then plotting log *P*(*k*)/log *k* against 1/log *k* (for networks of order *n* ≫ *k*). Extrapolating the data to *k* = ∞ using a linear or a quadratic regression gives the value of *γ* as the *y*-intercept of the graph. This method works well if *P*(*k*) scales with *k* as in Eq (1). However, strong corrections to the powerlaw behaviour may make the extrapolation difficult or inaccurate.

A second method to estimate *γ* is to note that if *γ* > 1, then for a fixed value of *α* > 0,

$$\begin{array}{c} \zeta (k)=\log P(\alpha \;k)-\log P(k)=-\gamma \log\alpha +o(1). \end{array}$$ (4)

Experimentation with numerical data shows that by plotting *ζ*(*k*) against $\frac{1}{k}\log k$ good results are obtained, and linear or quadratic regressions of *ζ*(*k*) against $\frac{1}{k}\log k$ can be used to estimate *γ*.

If it is assumed that *P*(*k*) is well approximated by Eq (1) for all *k* ≥ 1, then the average *connectivity* of a network of order *n* with average degree distribution proportional to *P*(*k*) = *C*_*o*_ *n*^−*γ*^ is given by

$$\begin{array}{ccc} {\left\langle k\right\rangle }_{n} & = & \frac{\sum_{k=1}^{n}k\;P\left(k\right)}{\sum_{k=1}^{n}P\left(k\right)}≃\frac{\int_{1}^{n}k\;P\left(k\right)\;dk}{\int_{1}^{n}P\left(k\right)\;dk}≃\left(\frac{\gamma -1}{\gamma -2}\right)\frac{n^{\gamma }-n^{2}}{n^{\gamma }-n}\\ & ≃ & \left\{ \begin{array}{cc} \left(\frac{\gamma -1}{2-\gamma }\right)n^{2-\gamma }, & \text{if}\;1<\gamma <2;\\ (\frac{\gamma -1}{\gamma -2}), & \text{if}\;\gamma >2. \end{array}\right. \end{array}$$ (5)

Observe that the asymptotic estimate is very poor if *γ* ≈ 2, and if *n* is small.

The cases *γ* = 1 and *γ* = 2 can also be determined; this gives

$$\begin{array}{c} {\left\langle k\right\rangle }_{n}≃\left\{ \begin{array}{cc} \frac{n}{\log n}, & \text{if}\;\gamma =1;\\ \log n, & \text{if}\;\gamma =2. \end{array}\right. \end{array}$$ (6)

The coefficient $\frac{\gamma -1}{\gamma -2}$ may be modifed if *P*(*k*) is not well approximated by the powerlaw decay for smaller values of *k* in Eq (1). These results, however, do show that the connectivity is a constant independent of *n* (for large *n*) if *γ* > 2.

The expected number of bonds in the network is given by $E_{n}=\frac{1}{2}n\;\;{\langle k\rangle }_{n}$. Assuming the powerlaw relation in Eq (1), it follows that

$$\begin{array}{c} E_{n}=\left\{ \begin{array}{cc} \frac{n^{2}}{2\log n}, & \text{if}\;\gamma =1;\\ \left(\frac{\gamma -1}{2(\gamma -2)}\right)n^{3-\gamma }, & \text{if}\;1<\gamma <2;\\ \frac{1}{2}n\log n, & \text{if}\;\gamma =2;\\ (\frac{\gamma -1}{2(\gamma -2)})n, & \text{if}\;\gamma >2. \end{array}\right. \end{array}$$ (7)

Of course, if *γ* < 1, then *E*_*n*_ = Θ(*n*^2^) and since a complete graph has $\frac{1}{2}n\left(n-1\right)$ bonds, this implies that these graphs are dense in the sense that ${lim inf}_{n\to \infty }\frac{1}{n^{2}}E_{n}>0$. For all values of *γ* ≥ 1 the above shows that ${lim sup}_{n\to \infty }\frac{1}{n^{2}}E_{n}=0$, and the graphs are sparse.

These results are useful in examining numerical data for scale-free networks. For example, *γ* can be estimated by examining degree sequences averaged over randomly sampled networks (from Eq (1)), or alternatively by using Eq (4). The connectivity 〈*k*〉_*n*_ approaches a constant if *γ* > 2 (as in Eq (5)) or grows as a powerlaw with *n* if *γ* < 2, and with logarithmic corrections if *γ* = 1 or *γ* = 2 (as in Eq (6)). Alternatively, the average size *E*_*n*_ (the number of bonds in a network of order *n*) can be considered, using the results in Eq (7).

## Mean field theory and scale-free networks

### Barabási-Albert networks and the Barabási-Albert algorithm

The Barabási-Albert algorithm is a recursive algorithm which grows networks (or clusters of nodes and bonds) from a seed node. This algorithm was introduced in reference [8] and reviewed in 2002 in a seminal paper [6], and its elementary move was inspired by processes underlying the (presumed) evolution of scale-free networks seen in the physical world. The elementary move is a preferential attachment of new nodes (and bonds) to hubs (nodes of high degree) in the network. The algorithm is initiated by a single node, and then new nodes and bonds are recursively attached, with new bonds preferentially attached to existing nodes of large degree.

A Barabási-Albert network of order *N* nodes is grown as follows:

**Barabási-Albert algorithm:**

1. Initiate the network with one node *x*_0_;
2. Suppose that the network consists of nodes {*x*_0_, *x*_1_, …, *x*_*n*−1_} of degrees {*k*_0_, *k*_1_, …, *k*_*n*−1_};
3. Append a new node *x*_*n*_ by executing step (a) or step (b):
  (a) With probability *p*: Select *x*_*j*_ uniformly and attach *x*_*n*_ to it by inserting the bond 〈*x*_*j*_ ∼ *x*_*n*_〉;
  (b) With default probability 1 − *p*: Attach *x*_*n*_ by adding bonds 〈*x*_*j*_ ∼ *x*_*n*_〉 independently with probability $\frac{k_{j}}{\sum_{j}k_{j}}$;
4. Repeat step 3 until a network of order *N* is grown.

Step 3(a) is a random attachment of a node and bond, and step 3(b) attaches a node with bonds *preferentially* to existing nodes of high degree. The algorithm has a single parameter *p*. If *p* = 1 then the algorithm grows acyclic (and connected) networks of order *N* (these are random trees).

On the other hand, if *p* = 0, then step 3(b) is executed on each iteration. New bonds are created with probabilities $q_{j}=\frac{k_{j}}{\sum_{j}k_{j}}$ for *j* = 0, 1, …, *n* − 1 when the *n*-th node is added. This shows that the expected number of bonds added in this step is on average ∑_*j*_ *q*_*j*_ = 1. That is, on average 1 bond is added in each iteration, and the average sum of degrees ∑_*j*_ *k*_*j*_ should be equal to 2*n* by handshaking after *n* iterations. This suggests that the algorithm grows a sparse graph with increasing *n*. However, since bonds are appended preferentially on growing hubs, the largest clusters in the network should be dominated by growing hubs.

For values of *p* ∈ (0, 1) the algorithm adds either (wih probability *p*) a single bond randomly, or it adds a collection of bonds (on average one bond) preferentially. This grows simple networks of order *N* and size *N* − 1, typically not connected unless acyclic.

**Fig 1:**
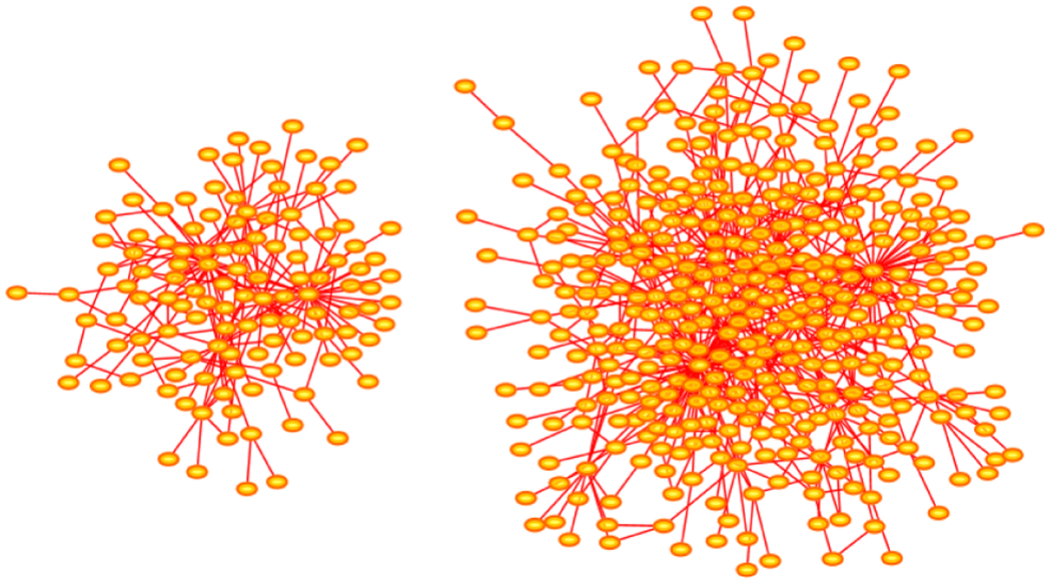
Barabási-Albert networks with *p* = 0. The network on the left was grown to order *n* = 122. It has 5 hubs of degrees {12, 17, 18, 19, 31} exceeding $\sqrt{122}$. The network on the right was grown to order *n* = 380. This network has 3 hubs of degrees {29, 47, 63} exceeding $\sqrt{380}$. The arrangement of nodes and bonds in these networks was created using the prefuse force directed lay-out in Cytoscape 3.4.0 [17].

In Fig 1 an example of a Barbasi-Albert network of order 122 with *p* = 0 is shown (left) and the right is a network of size 380. The appearance of hubs in these networks is clearly seen: In the network on the left there are 5 nodes of degrees exceeding $\sqrt{122}$, the largest of degree 31, and in the network on the right there are 3 hubs of degrees exceeding $\sqrt{380}$, the largest of degree 63.

#### Modified Barabási-Albert networks

Barabási-Albert networks are relatively sparse networks. A modification of the algorithm can be introduced to grow denser networks. For example, one may replace step 3(b) by

3(b) With default probability 1 − *p*: Attach *x*_*n*_ by adding bonds 〈*x*_*j*_ ∼ *x*_*n*_〉 with probability $q_{j}=\min\left\{\frac{\lambda \;k_{j}+A}{\sum_{j}k_{j}},1\right\}$ (where *λ* and *A* are non-negative parameters of the algorithm);

Since *k*_*j*_ ≪ ∑_*j*_ *k*_*j*_ in Barabási-Albert networks, one may assume that *λk*_*j*_ + *A* ≤ ∑_*j*_ *k*_*j*_ for values of *λ* and *A* which are not too large (and so *q*_*j*_ ≤ 1).

**Fig 2:**
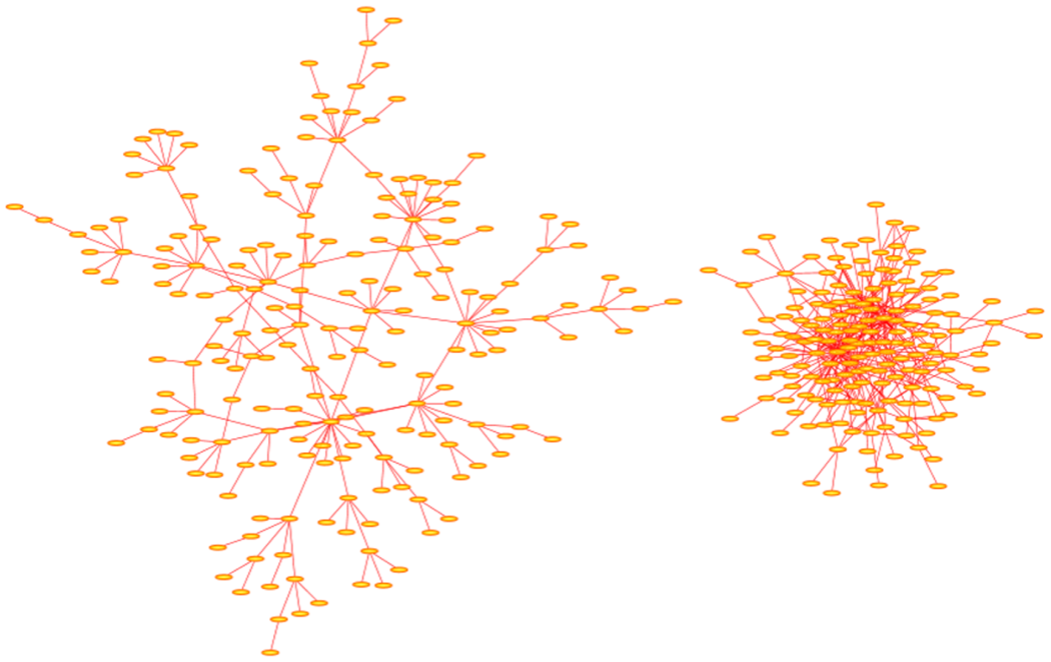
Modified Barabási-Albert networks. The network on the left was grown with *λ* = 0.1 to order *n* = 203. It has two hubs of degrees {15, 17} which exceed $\sqrt{203}$. The network on the right was grown with *λ* = 1.5 to order *n* = 172. This network contains hubs of degrees {15, 15, 16, 17, 19, 27, 33} exceeding $\sqrt{172}$. In both cases the algorithm was implemented with *p* = 0. The arrangement of nodes and bonds in these networks was created using the prefuse force directed lay-out in Cytoscape 3.4.0 [17].

In Fig 2 two examples of Modified Barabási-Albert networks are shown, one a sparse network with *λ* = 0.5, *A* = 0 and *p* = 0, and the second a denser network with *λ* = 2.0, *A* = 0 and *p* = 0. In both cases the algorithm was iterated 200 times; the sparse network has order 203 and two hubs of degrees {15, 17}, and the dense network has order 172 with seven hubs of degrees {15, 15, 16, 17, 19, 27, 33}.

#### Variant Barbasi-Albert networks

A variant Barbasi-Albert algorithm can be introduced by changing step 3(b) in the Barbasi-Albert algorithm to

**Fig 3:**
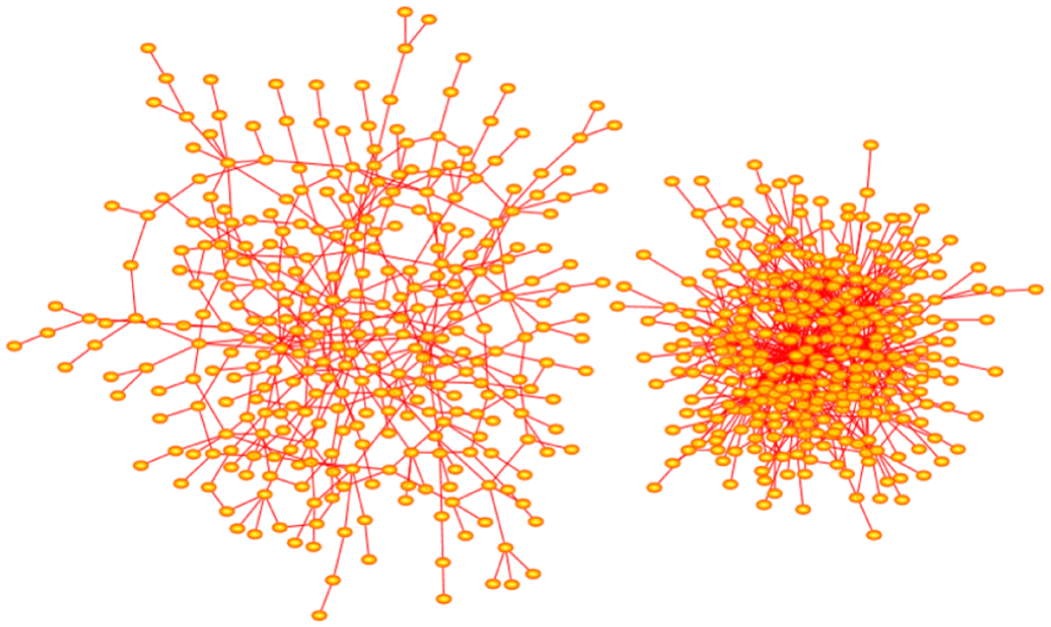
Variant Barabási-Albert networks. The network on the left was grown using *α* = 0.15 and *A* = 0 to a total to *n* = 327 nodes. This graph is very sparse, and none of its nodes qualify as hubs. The network on the right was grown to order *n* = 351 with *α* = 1.15 and *A* = 0. This is a dense network with several nodes qualifying as hubs of degrees {22, 24, 26, 42, 43, 116}. The arrangement of nodes and bonds in these networks was created using the prefuse force directed lay-out in Cytoscape 3.4.0 [17].

3(b) With default probability 1 − *p*: Attach *x*_*n*_ by adding bonds 〈*x*_*j*_ ∼ *x*_*n*_〉 with probability $q_{j}=\min\left\{\frac{k_{j}^{\alpha }+A}{\sum_{j}k_{j}},1\right\}$, (where *α* and *A* are non-negative parameters of the algorithm);

The effect of the parameter *α* is to increase the probability of adding bonds to the hubs of the network if *α* > 1, and to decrease this probability if *α* < 1. In the case that *α* > 1 networks dominated by a single very large hub are obtained (see Fig 3 (right network)), while networks with *α* < 1 are more sparse and not dominated by a few hubs (see Fig 3 (left network)). The left network in Fig 3 was grown by putting *α* = 0.15 and *A* = 0 and has order 327. None of the nodes in this network has degree which exceeds $\sqrt{327}$, and so none qualify as hubs. A denser network is obtained if *α* = 1.15 and *A* = 0, as shown in Fig 3 on the right. This network is dominated by hubs of degrees {22, 24, 26, 42, 43, 116} and has order 351.

#### Mean field theory for Modified Barabási-Albert networks

Let *k*_*j*_(*n*) be the degree of node *j* after *n* iterations. A mean field calculation of *k*_*j*_(*n*) is done by assuming that *k*_*j*_(*n*) is equal to its expected value for each *n*; that is, *k*_*j*_(*n*) = 〈*k*_*j*_(*n*)〉 for each *j* and *n*.

The modified Barabási-Albert algorithm appends bonds to a network of order *n* as follows: Step 3(a) is executed with probability *p*, and a bond (and the (*n* + 1)-th node) is appended with uniform probability on one of the *n* existing nodes. The probability that node *j* gets a bond in this way is $\frac{p}{n}$ and on average one bond is attached with probability *p*.

If step 3(b) is done instead, then the expected number of bonds added in the mean field is approximately $\sum_{j}\frac{\lambda \;k_{j}\left(n\right)+A}{\sum_{j}k_{j}\left(n\right)}=\lambda +\frac{nA}{\sum_{j}k_{j}\left(n\right)}$. The total number of bonds in the network is

$$\begin{array}{c} 2E_{n}=\sum_{j}k_{j}\left(n\right) \end{array}$$ (8)

by handshaking. Thus, the increment in the number of bonds when the next node is appended is

$$\begin{array}{c} \Delta E_{n}=p+\left(1-p\right)\lambda +\left(1-p\right)\frac{nA}{2E_{n}}. \end{array}$$ (9)

Approximate this by a differential equation

$$\begin{array}{c} 2E_{n}\frac{d}{dn}E_{n}=2\left(p+\left(1-p\right)\lambda \right)E_{n}+\left(1-p\right)nA. \end{array}$$ (10)

This can be solved to obtain

$$\begin{array}{c} E_{n}=\frac{n}{2}\left(\left(p+\left(1-p\right)\lambda \right)+\sqrt{{\left(p+(1-p)\lambda \right)}^{2}+2\left(1-p\right)A}\right)=C\;\;n, \end{array}$$ (11)

where *C* is a function of (*p*, *λ*, *A*) defined by this expression. Notice that *E*_*n*_ grows linearly in *n*, so that Barabási-Albert graphs will be necessarily sparse as *n* → ∞ (and by Eq (7) the scaling exponent is *γ* > 2).

With each iteration the mean field value of *k*_*j*_(*n*) (the degree of the *j*-th node after *n* iterations) increments by

$$\begin{array}{c} k_{j}\left(n+1\right)=k_{j}\left(n\right)+\frac{p}{n}+\frac{\left(1-p\right)(\lambda k_{j}\left(n\right)+A)}{2E_{n}} \end{array}$$ (12)

since 2*E*_*n*_ = ∑_*j*_ *k*_*j*_(*n*) = 2*Cn*, and since the probabilty of adding a bond to node *j* is $\frac{\lambda k_{j}\left(n\right)+A}{\sum_{j}k_{j}\left(n\right)}$. This can again be approximated by a differential equation: Take *n* → *t*, a continuous time variable, and let *k*_*j*_(*n*) → *k*_*j*_(*t*), the continuous mean field degree of node *j*. Then

$$\begin{array}{c} \frac{d}{dt}k_{j}\left(t\right)=\frac{p}{t}+\frac{\left(1-p\right)(\lambda k_{j}\left(t\right)+A)}{2Ct}. \end{array}$$ (13)

The initial condition is to assume that node *j* is added at time *t*_*j*_. Putting *A* = 0 and *λ* = 1 gives *C* = 1 and the equation

$$\begin{array}{c} \frac{d}{dt}k_{j}\left(t\right)=\frac{p}{t}+\frac{\left(1-p\right)k_{j}\left(t\right)}{2t} \end{array}$$ (14)

which was also derived in reference [9]. In this event the solution is $k_{j}\left(t\right)=\frac{1+p}{1-p}{\left(t/t_{j}\right)}^{(1-p)/2}-\frac{2p}{1-p}$ (assuming the initial condition *k*_*j*_(*t*_*j*_) = 1).

More generally, Eq (13) can be cast in the general form

$$\begin{array}{c} \frac{d}{dt}k_{j}\left(t\right)=\frac{Q}{t}+\frac{P}{t}k_{j}\left(t\right) \end{array}$$ (15)

where $Q=p+\frac{(1-p)A}{2C}$ and $P=\frac{(1-p)\lambda }{2C}$, with solution

$$\begin{array}{c} k_{j}\left(t\right)=\left(1+\frac{Q}{P}\right)\;{\left(t/t_{j}\right)}^{P}-\frac{Q}{P} \end{array}$$ (16)

using again the intial condition *k*_*j*_(*t*_*j*_) = 1.

The mean field degree distribution can be determined from this solution. The probability that node *j* has degree *k*_*j*_(*t*) smaller than *κ* at time *t* is denoted by *P*[*k*_*j*_(*t*) < *κ*]. Since *k*_*j*_(*t*) < *κ* if

$$\begin{array}{c} \left(1+\frac{Q}{P}\right){\left(t/t_{j}\right)}^{P}<\kappa \;\text{or,}\;\text{equivalently,}\;t_{j}>t{\left(\frac{Q/P+\kappa }{1+Q/P}\right)}^{-1/P}, \end{array}$$

this is also the probability $P[\left(t_{j}/t\right)>{\left(\frac{Q/P+\kappa }{1+Q/P}\right)}^{-1/P}]$. If the node *t*_*j*_ is chosen uniformly from the *n* available, then

$$\begin{array}{c} P\left[k_{j}\left(t\right)<\kappa \right]=P\left[\left(t_{j}/t\right)>{\left(\frac{Q/P+\kappa }{1+Q/P}\right)}^{-1/P}\right]=1-{\left(\frac{Q/P+\kappa }{1+Q/P}\right)}^{-1/P}. \end{array}$$ (17)

The mean field degree distribution is the derivative of this to *κ*:

$$\begin{array}{c} P\left(\kappa \right)=P\left[k_{j}\left(t\right)=\kappa \right]=\frac{\partial }{\partial \kappa }P\left[k_{n}\left(t\right)<\kappa \right]=\frac{{\left(P+Q\right)}^{1/P}}{{\left(P\kappa +Q\right)}^{1+1/P}}. \end{array}$$ (18)

For large *κ* this shows that the modified Barbasi-Albert network is scale-free with exponent

$$\begin{array}{ccc} \gamma & = & 1+\frac{1}{P}=1+\frac{2C}{(1-p)\lambda }\\ & = & 1+\frac{\left(\left(p+\left(1-p\right)\lambda \right)+\sqrt{{\left(p+(1-p)\lambda \right)}^{2}+2\left(1-p\right)A}\right)}{(1-p)\lambda }. \end{array}$$ (19)

Putting *A* = 0 gives the exponent

$$\begin{array}{c} \gamma =3+\frac{2p}{(1-p)\lambda }. \end{array}$$ (20)

This is the mean field exponent of a modified Barabási-Albert network. For small *λ* < 1 the exponent is large, indicating a network with few nodes (if any) of high degree. For large *λ* > 1, $\gamma ↘3^{+}$. This is a lower bound on *γ* for modified Barabási-Albert networks.

If *λ* = 1, then the exponent *γ* is given by

$$\begin{array}{c} \gamma =1+\frac{1}{1-p}+\frac{\sqrt{1+2(1-p)A}}{1-p}. \end{array}$$ (21)

In this model one similarly finds that *γ* ≥ 3, and in fact, if *p* = 0, then $\gamma =2+\sqrt{1+2A}$. The parameter *A* may be used to tune the exponent *γ* for any given *p*.

If both *λ* = 1 and *A* = 0, then the known expression for *γ* for Barabási-Albert networks is recovered, namely

$$\begin{array}{c} \gamma =\frac{3-p}{1-p}. \end{array}$$ (22)

Notice that *γ* ≥ 3 and that *γ* = 3 if *p* = 0 [9].

The connectivity of modified Barabási-Albert networks is given by

$$\begin{array}{c} {\left\langle k\right\rangle }_{n}≃\frac{\int_{1}^{n}k\;P\left(k\right)\;dk}{\int_{1}^{n}P\left(k\right)\;dk}≃\frac{2C}{2C-(1-p)\lambda }, \end{array}$$ (23)

where $2C=(\left(p+\left(1-p\right)\lambda \right)+\sqrt{{\left(p+(1-p)\lambda \right)}^{2}+2\left(1-p\right)A})$. Since $2-\gamma =1-\frac{1}{P}$, Eq (5) gives ${\langle k\rangle }_{n}≃\frac{1}{1-P}$. Inserting the value of *P* gives the result above as well.

**Fig 4:**
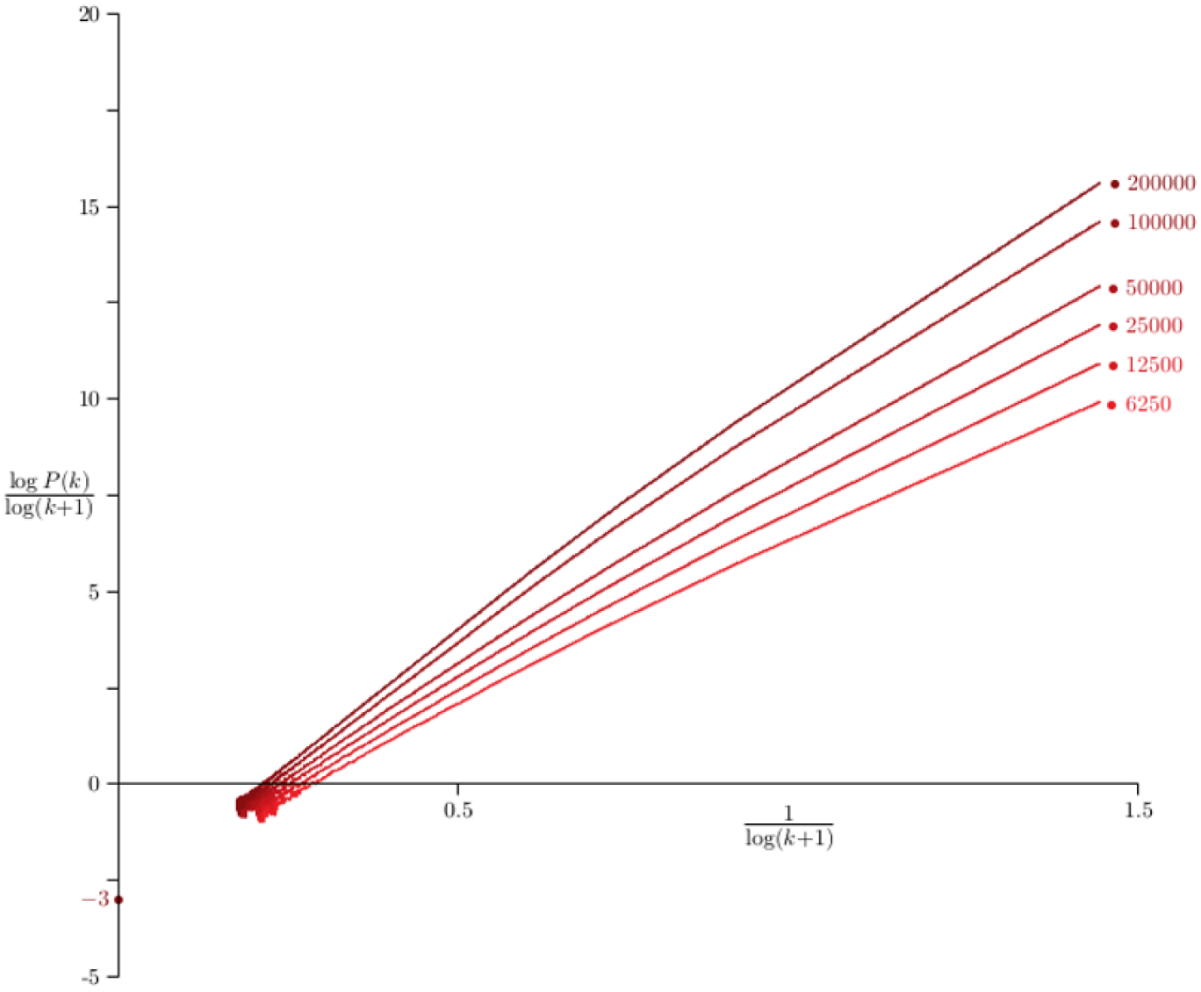
Scaling of Barabási-Albert networks with *p* = 0. Data on networks generated by the Barabási-Albert algorithm with *p* = 0. In each case 100 networks were grown and the average degree sequence *P*_*n*_(*k*) computed. The curves above are plots of log *P*_*n*_(*k*)/log(*k* + 1) against 1/log(*k* + 1) for *n* ∈ {6250, 12500, 25000, ⋯, 200000}. Least squares fit to the data using a quadratic model gives the *y*-intercepts which averages to 3.026. This is very close to the value *γ* = 3 predicted for the scaling exponent in this model by the mean field approach.

In the Fig 4 the probability *P*(*k*), that the degree of a Barabási-Albert network is equal to *k*, is examined by plotting log *P*(*k*)/log(*k* + 1) against 1/log(*k* + 1) where *P*(*k*) was estimated for values *n* ∈ {6250, 12500, 25000, 50000, 100000, 200000} and for *p* = 0. The curves should intersect the vertical axis at −*γ*. Least squares fit of the data to quadratic curves gives 6 estimates for *γ*, which average to *γ* = 3.026 ± 0.076, very close to the theoretical value *γ* = 3 from Eq (20) (for *p* = 0 and *λ* = 1).

Data collected for the same values of *n* and for *p* = 0.5 cannot be successfully analysed by regressions with quadratic curves, but cubic curves give the average value *γ* = 5.161 ± 0.068, which are not equal to but still fairly well approximated by *γ* = 5 predicted by Eq (20) for *p* = 0.5 and *γ* = 1.

When *p* = 0.8 the plots are strongly curved and extrapolation to estimate *γ* is more difficult. In this case a different approach is needed. Putting $\alpha =\frac{1}{2}$ in Eq (4) gives

$$\begin{array}{c} \log P\left(k\right)-\log P\left(\frac{1}{2}k\right)=-\gamma \log2+o\left(1\right) \end{array}$$ (24)

so that a plot of $\zeta \left(k\right)=(\log P\left(k\right)-\log P\left(\frac{1}{2}k\right))/\log2\to -\gamma$ as *k* → ∞. That is, plotting *ζ*(*k*) against $\frac{1}{k}$ gives a curve with *y*-intercept equal to −*γ*. Better results are obtained when plotting against $\frac{1}{k}\log k$. In this case a linear extrapolation gives *γ* = 11.67 ± 0.41 and a quadratic extrapolation gives *γ* = 11.6 ± 2.6. These results are close to the mean field prediction *γ* = 11 for *p* = 0.8. Incidently, if *p* = 0.5 then this kind of analysis show that *γ* = 5.47 ± 0.14 (linear extrapolation) or *γ* = 4.4 ± 1.0 (quadratic extrapolation), and if *p* = 0, then the results are *γ* = 3.088 ± 0.022 (linear extrapolation) and *γ* = 2.86 ± 0.18 (quadratic extrapolation).

If *λ* = 2 and *p* = *A* = 0 then the algorithm grows modified Barabási-Albert networks with *γ* = 3 (the mean field estimate given by Eq (19)). Estimating *γ* by plotting *ζ*(*k*) against $\frac{1}{k}\log k$ gives the estimate *γ* = 3.019 ± 0.098 (linear extrapolation) and *γ* = 2.62 ± 0.33 (quadratic extrapolation).

The connectivity of Modified Barabási-Albert networks should converge quickly to a constant with increasing *n* (by Eq (5)) since *γ* > 2. Computing it for Barabási-Albert networks (with *λ* = 1 and *A* = 0) gives 〈*k*〉_*n*_ ≈ 3.16 for *p* = 0, 〈*k*〉_*n*_ ≈ 2.28 for *p* = 0.5 and 〈*k*〉_*n*_ ≈ 2.08 for *p* = 0.8, and for *n* = 12500. Increasing *n* does not change these results.

#### Mean field theory for Variant Barabási-Albert networks

In this model the increment in the number of bonds when the (*n* + 1)-th node is appended is given by

$$\begin{array}{c} \Delta E_{n}=p+\frac{\left(1-p\right)\left(\sum_{j}{\left(k_{j}\left(n\right)\right)}^{\alpha }+A\right)}{2\;E_{n}}. \end{array}$$ (25)

Approximating this with a differential equation gives

$$\begin{array}{c} 2E_{n}\frac{d}{dn}E_{n}=2pE_{n}+\left(1-p\right)nA+\left(1-p\right)\sum_{j}{\left(k_{j}\left(n\right)\right)}^{\alpha }. \end{array}$$ (26)

The right hand side can be approximated as follows: For *α* > 1 the algorithm should grow dense networks with nodes of high degree. Assuming that *k*_*j*_(*n*) ≈ *k*_ℓ_(*n*) for all ℓ shows that $\sum_{j}{\left(k_{j}\left(n\right)\right)}^{\alpha }\approx n{\left(k_{j}\left(n\right)\right)}^{\alpha }\approx n{\left(\frac{1}{n}\sum_{j}k_{j}\left(n\right)\right)}^{\alpha }=n^{1-\alpha }{\left(2E_{n}\right)}^{\alpha }$. Using this approximation gives

$$\begin{array}{c} 2E_{n}\frac{d}{dn}E_{n}\approx 2pE_{n}+\left(1-p\right)nA+\left(1-p\right)n^{1-\alpha }{\left(2E_{n}\right)}^{\alpha }. \end{array}$$ (27)

If *A* = *p* = 0, then the differential equation can be solved directly to obtain *E*_*n*_ ≃ 2^(*α*−1)/(2−*α*)^ *n*, provided that *α* > 1. This shows that *E*_*n*_ is linear in *n*, which may be expected if *α* is not too much larger than 1.

Numerical experimentation shows that *E*_*n*_ grows linearly in *n* for values of *α* not too much larger than 1. For example, if *p* = 0.5, *A* = 1 and *α* = 1 then $\frac{1}{n}E_{n}\to 1.207\ldots$, if *α* = 1.5 then $\frac{1}{n}E_{n}\to 1.539\ldots$, but if *α* = 2 then $\frac{1}{n}E_{n}$ increases slowly with *n*. Similarly, if *p* = 0, and *A* = 1, then, if *α* = 1, $\frac{1}{n}E_{n}\to 1.366\ldots$, and if *α* = 1.5, $\frac{1}{n}E_{n}\to 2.399\ldots$, but if *α* = 2 then $\frac{1}{n}E_{n}$ increases slowly with *n* and for even larger values of *n* this growth accelerates.

The recurrence for the degree of the *j*-th node may be approximated by a differential equation similar to Eq (13): Assuming that *E*_*n*_ = *Dn*^*β*^, replacing *n* → *t* (a continuous time variable), gives the recurrence

$$\begin{array}{c} k_{j}\left(t+1\right)=k_{j}\left(t\right)+\frac{p}{t}+\frac{\left(1-p\right){(\left(k_{j}\left(t\right)\right)}^{\alpha }+A)}{2Dt^{\beta }}. \end{array}$$ (28)

This can be approximated by the differential equation

$$\begin{array}{c} \frac{d}{dt}k_{j}\left(t\right)=\frac{p}{t}+\frac{\left(1-p\right){(\left(k_{j}\left(t\right)\right)}^{\alpha }+A)}{2Dt^{\beta }}. \end{array}$$ (29)

If *α* = 1 and *β* = 1 then the solution of this equation gives the Barabási-Albert case with *γ* = 3. Proceed by considering the case *A* = *p* = 0 and the initial condition *k*_*j*_(*t*_*j*_) = 1. Assume that *α* = 1 + *ϵ*. Then the equation becomes

$$\begin{array}{c} \frac{2Dt^{\beta }}{k_{j}\left(t\right)}\;\frac{d}{dt}k_{j}\left(t\right)={\left(k_{j}\left(t\right)\right)}^{\epsilon }. \end{array}$$ (30)

A perturbative approach for small *ϵ* can be done by expanding ${\left(k_{j}\left(t\right)\right)}^{\epsilon }=\text{exp}\left(\epsilon \log k_{j}\left(t\right)\right)=1+\epsilon \log k_{j}\left(t\right)+\frac{1}{2}\epsilon^{2}\log^{2}k_{j}\left(t\right)+\cdots$. Truncating this at *O*(*ϵ*^2^) and putting *g*(*t*) = log *k*_*j*_(*t*) gives the differential equation

$$\begin{array}{c} 2Dt^{\beta }\frac{d}{dt}g\left(t\right)=1+\epsilon g\left(t\right)+\frac{1}{2}\epsilon^{2}g^{2}\left(t\right). \end{array}$$ (31)

Using the initial condition *g*(*t*_*j*_) = log *k*_*j*_(*t*_*j*_) = 0 the solution of this equation is

$$\begin{array}{c} \epsilon \;g\left(t\right)=\left\{ \begin{array}{cc} -1+\tan(\frac{\pi }{4}+\frac{\epsilon }{4D}\log\left(\frac{t}{t_{j}}\right)), & \;\text{if}\;\beta =1;\\ -1+\tan(\frac{\pi }{4}+\frac{\epsilon }{4D(\beta -1)}\left(t_{j}^{1-\beta }-t^{1-\beta }\right)), & \;\text{if}\;\beta >1. \end{array}\right. \end{array}$$ (32)

In the case *β* > 1 suppose that *δ* = *β* − 1 and that *δ* is small. Then approximate

$$\begin{array}{c} t_{j}^{1-\beta }-t^{1-\beta }=e^{-\delta \log t_{j}}-e^{-\delta \log t}\approx \delta \log\left(\frac{t}{t_{j}}\right)-\frac{1}{2}\delta^{2}\log\left(\frac{t}{t_{j}}\right)\log\left(tt_{j}\right)+O\left(\delta^{3}\right). \end{array}$$

With this approximation the solution for *g*(*t*) above can be expanded in *ϵ* and *δ* to give the first order approximations

$$\begin{array}{c} g\left(t\right)≃\left\{ \begin{array}{cc} \frac{1}{2D}\log\left(\frac{t}{t_{j}}\right)+\frac{\epsilon }{8D^{2}}\log^{2}\frac{t}{t_{j}}, & \;\text{if}\;\beta =1;\\ \frac{1}{2D}\log\left(\frac{t}{t_{j}}\right)+\frac{\epsilon }{8D^{2}}\log^{2}\frac{t}{t_{j}}-\frac{\delta }{4D^{2}}\left(D\log^{2}\left(\frac{t}{t_{j}}\right)+\log t_{j}\;\log\left(\frac{t}{t_{j}}\right)\right), & \;\text{if}\;\beta >1. \end{array}\right. \end{array}$$

Proceed by solving the above quadratics for $\log(\frac{t}{t_{j}})$ in terms of *g*(*t*). Expand the solution in *ϵ* and *δ* and keep only the first few terms. In the case that *β* = 1 this gives

$$\begin{array}{c} \log\left(\frac{t}{t_{j}}\right)\approx 2D\;g\left(t\right)-\epsilon D\;g^{2}\left(t\right). \end{array}$$ (33)

Since *g*(*t*) = log *k*_*j*_(*t*), the probability that *k*_*j*_(*t*) < *κ* is given by

$$\begin{array}{c} P\left[k_{j}\left(t\right)<\kappa \right]=P\left[\frac{t_{j}}{t}>\kappa^{\epsilon D\log\kappa -2D}\right]\approx 1-\kappa^{\epsilon D\log\kappa -2D}. \end{array}$$ (34)

Taking the derivative to *κ* gives the distribution function in the case that *β* = 1:

$$\begin{array}{c} P\left(k\right)∼D\left(2-D\epsilon \log k\right)\;k^{-1-2D+D\epsilon \;\log k}. \end{array}$$ (35)

These networks are thus not scale-free. For small values of *k* the log *k* terms are slowly varying, and the networks will appear to be scale-free with *γ* = 1 + 2*D*. However, with increasing *k* the exponent reduces in value and the connectivity of the network will become dependent on *k* in the way seen in Eq (5) for small values of *γ*.

Notice that if *D* = 1 and *ϵ* = 0 (or *α* = 1), then the above reduces to *P*(*k*) ∼ *k*^−3^, as expected for Barabási-Albert networks.

If *β* > 1, then a similar approach to the above may be considered. Solving the expression for *g*(*t*) above for $\log(\frac{t}{t_{j}})$ and keeping only terms to *O*(*ϵ*) and *O*(*δ*) gives

$$\begin{array}{c} \log\left(\frac{t}{t_{j}}\right)\approx 2D\;g\left(t\right)-\epsilon D\;g^{2}\left(t\right)+\delta \left(2D^{2}g^{2}\left(t\right)+g\left(t\right)\log t_{j}\right). \end{array}$$ (36)

This shows that

$$\begin{array}{ccc} P(k_{j}\left(t\right)<\kappa ) & = & P(\frac{t_{j}}{t}>\kappa^{\epsilon D\log\kappa -2D-2D^{2}\delta \log\kappa -\delta \log t_{j}})\\ & \approx & 1-\kappa^{\epsilon D\log\kappa -2D-2D^{2}\delta \log\kappa -\delta \log t_{j}}. \end{array}$$

This shows that

$$\begin{array}{c} P\left(k\right)∼\left(2D\left(1+2D\delta \log k-\epsilon \log k\right)\right)\;k^{-1-2D-\delta \log t_{j}-D\left(2D\delta -\epsilon \right)\log k}. \end{array}$$ (37)

This gives an effective exponent *γ*_*k*_ = 1 + 2*D* + *δ* log *t*_*j*_ + *D*(2*Dδ* − *ϵ*)log *k* which decreases in size if 2*Dδ* − *ϵ* < 0 and increases in size if 2*Dδ* − *ϵ* > 0. Since *δ* = *β* − 1 and *ϵ* = *α* − 1, and for small *α* numerical simulations show that *β* ≈ 1, it is normally the case that 2*Dδ* − *ϵ* < 0. This means that the networks will first appear scale-free with constant connectivity until *k* becomes large enough in which case the connectivity will increase with *k*, as seen above.

#### Numerical results on Variant Barabási-Albert networks

**Fig 5:**
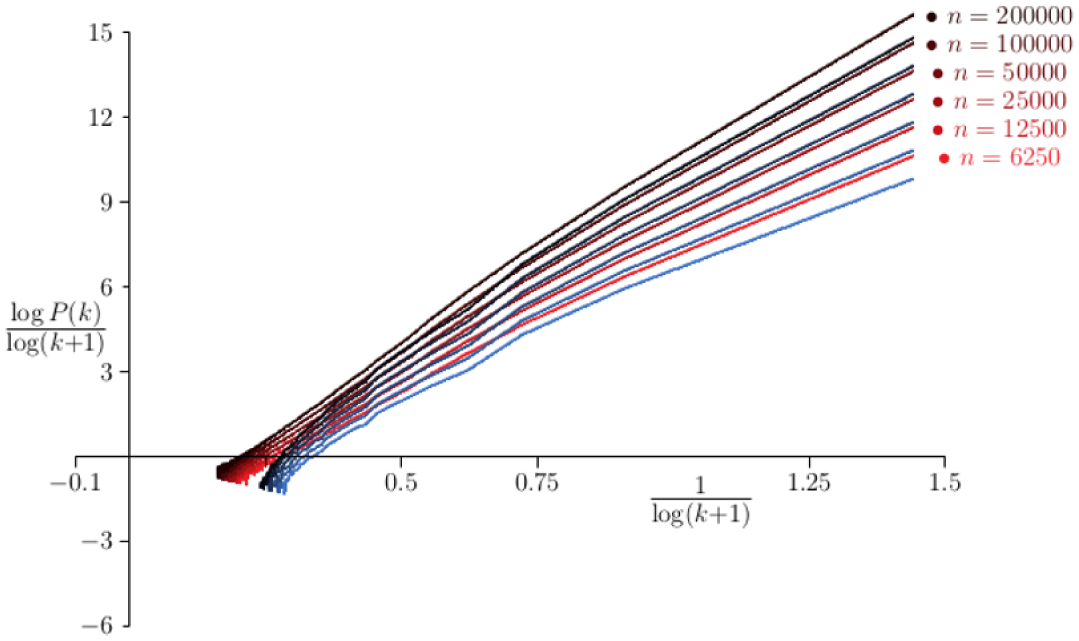
Variant Barabási-Albert networks with *p* = 0. Data on networks generated by the Variant Barabási-Albert algorithm with *p* = 0 and *α* = 1.1 (red curves) and *α* = 0.5 (blue curves). In each case 100 networks were grown and the average degree sequence *P*_*n*_(*k*) computed. The curves above are plots of log *P*_*n*_(*k*)/log(*k* + 1) against 1/log(*k* + 1) for *n* ∈ {6250, 12500, 25000, ⋯, 200000}.

In Fig 5 data for networks with *p* = 0 and *α* = 1.1 and *α* = 0.5 is shown. Since *α* = 1.1 is still very close to 1, the results above show that these networks should still appear scale-free, and with connectivity a constant. This is indeed the case. For *n* = 6250 the data gives 〈*k*〉_*n*_ = 3.149, and increasing *n* to *n* = 200000 gives 〈*k*〉_*n*_ = 3.176. That is, the connectivity of the networks are insensitive to *n* over this range. Least squares fits to the curves with quadratic polynomials in order to determinate the value of *γ* give the average *γ* = 2.857 ± 0.068. This result is consistent with a constant value of the connectivity of networks of these size ranges. With increasing *n*, it is expected that *γ* will decrease in value (that is, the value given here is an effective value), and eventually, the connectivity will start to increase.

Networks generated with *p* = 0 and *α* = 0.5 turned out to be sparse with low connectivity. For example, for *n* = 100000, the connectivity is 〈*k*〉_*n*_ = 1.036 and this decreases even further for *n* = 200000, where 〈*k*〉_*n*_ = 1.020. Attemps to extract an exponent *γ* from the data for these networks were not succesful, the regressions did not settle on a value, but are strongly dependent on *n*. Notice that the mean field analysis above does not apply to networks with *α* < 1.

Putting *α* = 2 gives networks with average connectivity which increases with *n*. For example, if *n* = 100, then 〈*k*〉_*n*_ = 43, for *n* = 500, 〈*k*〉_*n*_ = 260 and for *n* = 1000, 〈*k*〉_*n*_ = 527. On the other hand, if $\alpha =\frac{3}{2}$, then 〈*k*〉_*n*_ = 3.08 if *n* = 100, 〈*k*〉_*n*_ = 3.27 if *n* = 500, and 〈*k*〉_*n*_ = 3.31 if *n* = 1000, and it appears that for small values of *n* the connectivity does not change quickly with increasing *n*.

### Duplication-Divergence networks

Biological models of protein evolution are usually presented in terms of two processes, namely (1) a *duplication event* involving a gene sequence in DNA, and (2) a *(random) mutation* of duplicated genes which then drift from one another in genetic space [18–20]. The mutations of duplicated and mutated genes change the proteome and the network of protein interactions: If the protein is self-interacting, then the duplicated proteins interact, and the mutated genes code for proteins with altered interactions (some gained, others weakened or lost) with other proteins.

The Duplication-Divergence algorithm models these processes in order to grow a network, and was used in order to estimate the rates of duplication and mutation in the protein interaction networks [11]. There is a rich and large literature reporting on modeling protein interaction networks using models which include processes of duplication and divergence [21–24].

Since proteomic networks appear to be scale-free [25, 26], it seems likely that duplication and divergence processes should grow scale-free networks and that this should also be seen in computer algorithms which grow networks using duplication and divergence elementary moves. Duplication can be implemented by selecting nodes and duplicating them, and their incident bonds, in a network. Divergence is implemented by altering the bonds incident on particular nodes, namely either by deleting, adding or moving bonds. In the Duplication-Divergence algorithm these moves are implemented by selecting nodes uniformly for duplication to progenitor-progeny pairs, and by deleting bonds incident to either the progenitor node or its progeny. Notice that since nodes of high degree have a larger probability of being adjacent to a node selected for duplication, these nodes have a larger probability of receiving new bonds in the duplication process—in this way there are events of preferential attachment in this algorithm [15, 16].

**Fig 6:**
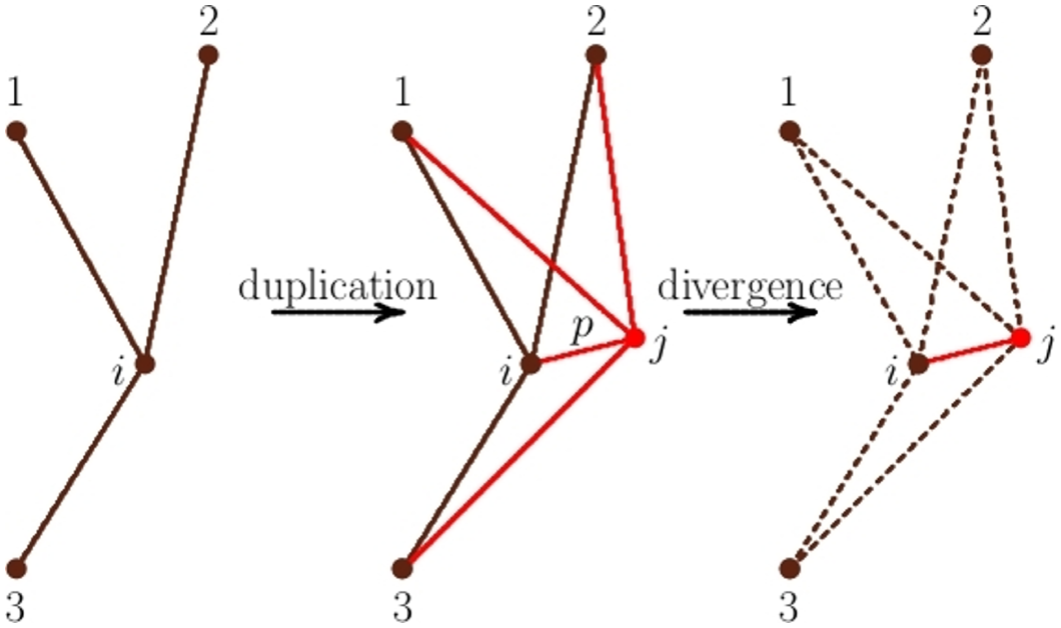
The Duplication-Divergence algorithm. Duplication-Divergence iterations: A node *i* and its incident bonds are duplicated to create a node *j* with its incident bonds. The bond 〈*i* ∼ *j*〉 is added with probability *p*. In the divergence step one of the pair of bonds (〈*i* ∼ *m*〉, 〈*j* ∼ *m*〉) is deleted with probability *q*, for each value of *m* ∈ {1, 2, 3}.

The basic elementary move of the Duplication-Divergence algorithm is illustrated in Fig 6.

The algorithm is implemented as follows.

**Duplication-Divergence algorithm:**

1. Initiate the network with one node *x*_0_ and apply the following steps iteratively;
2. Duplication: Choose a node *υ* uniformly and duplicate by creating node *υ*′;
3. For all bonds 〈*w* ∼ *υ*〉 incident with *υ*, add the bonds 〈*w* ∼ *υ*′〉;
4. With probability *p* add the bond 〈*υ* ∼ *υ*′〉;
5. Divergence: delete one bond of the pair {〈*w* ∼ *υ*〉, 〈*w* ∼ *υ*′〉} incident with *υ* or with its duplicated node *υ*′ with probability *q* (for each *w* adjacent to both *υ* and *υ*′ independently);
6. Stop the algorithm when a network of order *N* is grown.

The algorithm has two parameters (*p*, *q*).

The parameter *p* is the probability that the protein corresponding to the progenitor node *υ* is self-interacting. If it is (with probability *p*) then the bond 〈*υ* ∼ *υ*′〉 is added to the network and it represents the interaction between *υ* and *υ*′.

The parameter *q* controls the model of divergence in this algorithm. As *υ* and *υ*′ diverge from one another, one bond in each pair of bonds incident with *υ* and *υ*′ is lost independently, with probability *q*. The result is that the network mutates as bonds (interactions) are lost (while they are created by the duplication process).

A slightly modified algorithm is found by changing step 5 in the algorithm to find a modification of the Duplication-Divergence algorithm which assumes that one of the duplicated pair mutates, while the other remains stable.

5 Divergence: Consider all bonds 〈*w* ∼ *υ*′〉 incident with the duplicated node *υ*′ and delete these independently with probability *q*.

The Duplication-Divergence algorithm tends to grow disconnected networks, while the Modifed Duplication-Divergence algorithm is more likely to grow networks with a single component (that it, connected networks).

#### Mean field theory for Duplication-Divergence networks

Let *k*_*j*_(*n*) be the degree of node *j* after *n* iterations. The algorithm appends nodes by duplicating them (the probability that a node *υ* is duplicated in a network of order *n* is $\frac{1}{n}$), adds bonds by inserting a bond between a node and its duplicate with probability *p*, and remove bonds by selecting one bond between node-duplicate pairs and other nodes independently and deleting it with probability *q*. Let 2*E*_*n*_ = ∑_*j*_ *k*(*n*) be twice the total number of bonds after *n* iterations. Then, if *k*_*j*_(*n*) is the degree of node *j* at time *n*, and node *j* is duplicated, the number of bonds in the network *E*_*n*_ increases in the mean field by

$$\begin{array}{c} E_{n+1}=E_{n}+p+k_{j}\left(n\right)-q\;k_{j}\left(n\right). \end{array}$$ (38)

This follows since *k*_*j*_(*n*) bonds are created in the duplication move in the mean field, and another bond is created between the *j*-th node and its duplicate with probability *p*. The number of deleted bonds in the mean field is *qk*_*j*_(*n*).

Notice that 2*E*_*n*_ = ∑_*j*_ *k*_*j*_(*n*) = *na*_*n*_ where *a*_*n*_ = 〈*k*_*j*_(*n*)〉 is the average degree. In the mean field approximation one substitutes *k*_*j*_(*n*) in the recurrence (38) by its network average *a*_*n*_. Then Eq (38) can be casted as a recurrance for *a*_*n*_:

$$\begin{array}{c} \left(n+1\right)\;a_{n+1}=n\;a_{n}+2p+2\left(1-q\right)\;a_{n}. \end{array}$$ (39)

Let *n* → *t*, where *t* is a continuous time variable, and approximate this recurrence by the differential equation

$$\begin{array}{c} t\frac{d}{dt}a_{t}=2p+\left(1-2q\right)\;a_{t}. \end{array}$$ (40)

The initial condition is *a*_1_ = 1, and this has solution

$$\begin{array}{c} a_{t}=\frac{1-2(q-p)}{1-2q}\;t^{1-2q}-\frac{2p}{1-2q}. \end{array}$$ (41)

Since $E_{n}≃\frac{1}{2}n\;a_{n}$, it follows that

$$\begin{array}{c} E_{n}=\frac{1-2(q-p)}{2(1-2q)}\;n^{2(1-q)}-\frac{pn}{1-2q}. \end{array}$$ (42)

Comparison to Eq (7) shows that, if $q<\frac{1}{2}$,

$$\begin{array}{c} \gamma =1+2q. \end{array}$$ (43)

In this case *E*_*n*_ = *O*(*n*^2(1−*q*)^) + *O*(*n*) and that while 2(1 − *q*) > 1, the term *O*(*n*) is a strong correction to the growth in *E*_*n*_ for even large values of *n*. In other words, the degree distribution *P*(*k*) of the network will be strongly corrected from the powerlaw distribution in Eq (1).

If $q=\frac{1}{2}$, then by solving Eq (40), *a*_*t*_ = 1 + 2*p* log *t* (so that *a*_1_ = 1). Since $E_{n}=\frac{1}{2}n\;a_{n}$, this shows that

$$\begin{array}{c} E_{n}=\frac{1}{2}n+pn\log n,\;\text{if}\;q=\frac{1}{2}. \end{array}$$ (44)

In this case *γ* = 2 by Eq (7), but notice the subtle domination of the *n* log *n* term. In numerical work this will be very hard to see.

The case $q>\frac{1}{2}$ is considered by noting that $a_{t}≃\frac{2p}{2q-1}$ as *t* → ∞. This shows that

$$\begin{array}{c} E_{n}≃\frac{p\;n}{2q-1},\;\text{if}\;q>\frac{1}{2}. \end{array}$$ (45)

This shows that *γ* ≥ 2 by Eq (7).

Putting the above together gives

$$\begin{array}{c} \gamma \;\left\{ \begin{array}{cc} =1+2q, & \text{if}\;q\leq \frac{1}{2};\\ \geq 2, & \text{if}\;q>\frac{1}{2}. \end{array}\right. \end{array}$$ (46)

with a logarithmic correction if $q=\frac{1}{2}$.

Comparing the coefficient in Eq (7) with Eq (45) gives a refined estimate $\gamma =1+\frac{2p}{1+2p-2q}\geq 2$, provided that 2*q* < 1 + 2*p*. For example, if *q* = 0.75 then *p* > 0.25. However, numerical work shows this estimate to be too small, and estimating *γ* in this regime for this model remains an open question.

The power law decrease in *P*(*k*) in Eq (1) is only asymptotic for this algorithm; and there should be corrections in particular for $q<\frac{1}{2}$. From the results above the average connectivity can be computed: Since $E_{n}=\frac{1}{2}n\;\langle k_{j}\left(n\right)\rangle$,

$$\begin{array}{c} {\left\langle k\right\rangle }_{n}≃\left\{ \begin{array}{cc} \frac{1-2(q-p)}{1-2q}\;n^{1-2q}-\frac{2p}{1-2q}, & \text{if}\;q<\frac{1}{2};\\ 2p\log n+1, & \text{if}\;q=\frac{1}{2};\\ \text{Constant}, & \text{if}\;q>\frac{1}{2}. \end{array}\right. \end{array}$$ (47)

From these results *P*(*k*) can be calculated. Since ${\langle k\rangle }_{n}≃\int_{1}^{n}k\;P\left(k\right)\;dk$, it follows that $\frac{d}{dn}{\langle k\rangle }_{n}=n\;P\left(n\right)$. Thus, using this approach gives

$$\begin{array}{c} P\left(k\right)∼\left\{ \begin{array}{cc} \left(1-2\left(q-p\right)\right)\;k^{-1-2q}, & \text{if}\;q<\frac{1}{2};\\ 2p\;k^{-2}, & \text{if}\;q=\frac{1}{2};\\ C_{0}\;k^{-\gamma }, & \text{if}\;q>\frac{1}{2}, \end{array}\right. \end{array}$$ (48)

where the case $q>\frac{1}{2}$ is unknown since the dependence of the exponent *γ* on the parameters (*p*, *q*) is not known. Notice the change in behaviour at the critical value $q=\frac{1}{2}$; this was already observed numerically in reference [11].

The modified Duplication-Divergence algorithm has the same recurrence (41), and so the values for *γ* and relations for 〈*k*〉_*n*_ and *P*(*k*) remain unchanged for this algorithm. Notice that this implementation preserves the degree of the selected node, and tends to give a duplicated node with lower degree (while the (unmodified) implementation tends to lower the degrees of both the selected and duplicated nodes). As a result, networks generated with the modified algorithm have, on average, more nodes of degree equal to one (and so appear more tree-like).

#### Numerical results on Duplication-Divergence networks

**Fig 7:**
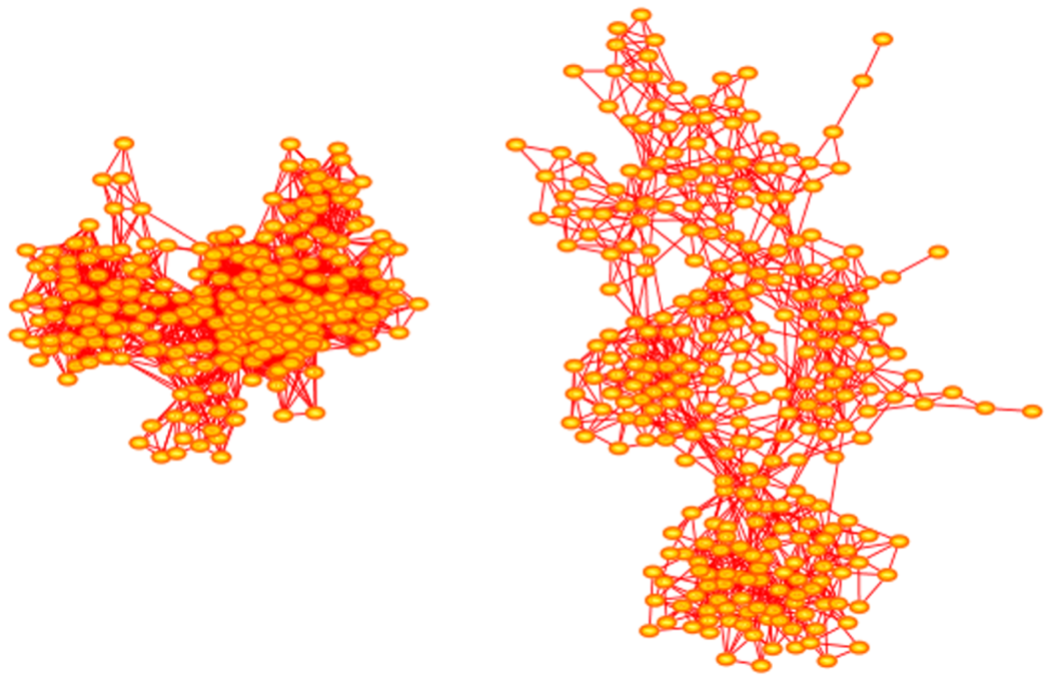
Duplication-Divergence network. The network on the left is a network generated with *p* = 1 and *q* = 0.40. It has order 300 and it has 114 nodes with degrees exceeding $\sqrt{300}$ and so qualify as hubs. The largest few of these hubs have degrees {43, 45, 47, 47, 50}. The network on the right is similarly a network generated with *p* = 1 and *q* = 0.60. It is more extended but has only one node of degree equal to one. Its order is 300, and it has 5 nodes of degrees {18, 18, 19, 20, 23} which qualify as hubs. Networks generated with the Modified Duplication-Divergence algorithm have a similar appearance, with the exception that more nodes of degree 1 are seen. The arrangement of nodes and bonds in these networks was created using the prefuse force directed lay-out in Cytoscape 3.4.0 [17].

In Fig 7 two networks grown with the Duplication-Divergence algorithm are shown. Both networks were grown with *p* = 1 and have order 300. The network on the left was grown with divergence parameter *q* = 0.4, and that on the right, with the higher mutation rate *q* = 0.6.

**Fig 8:**
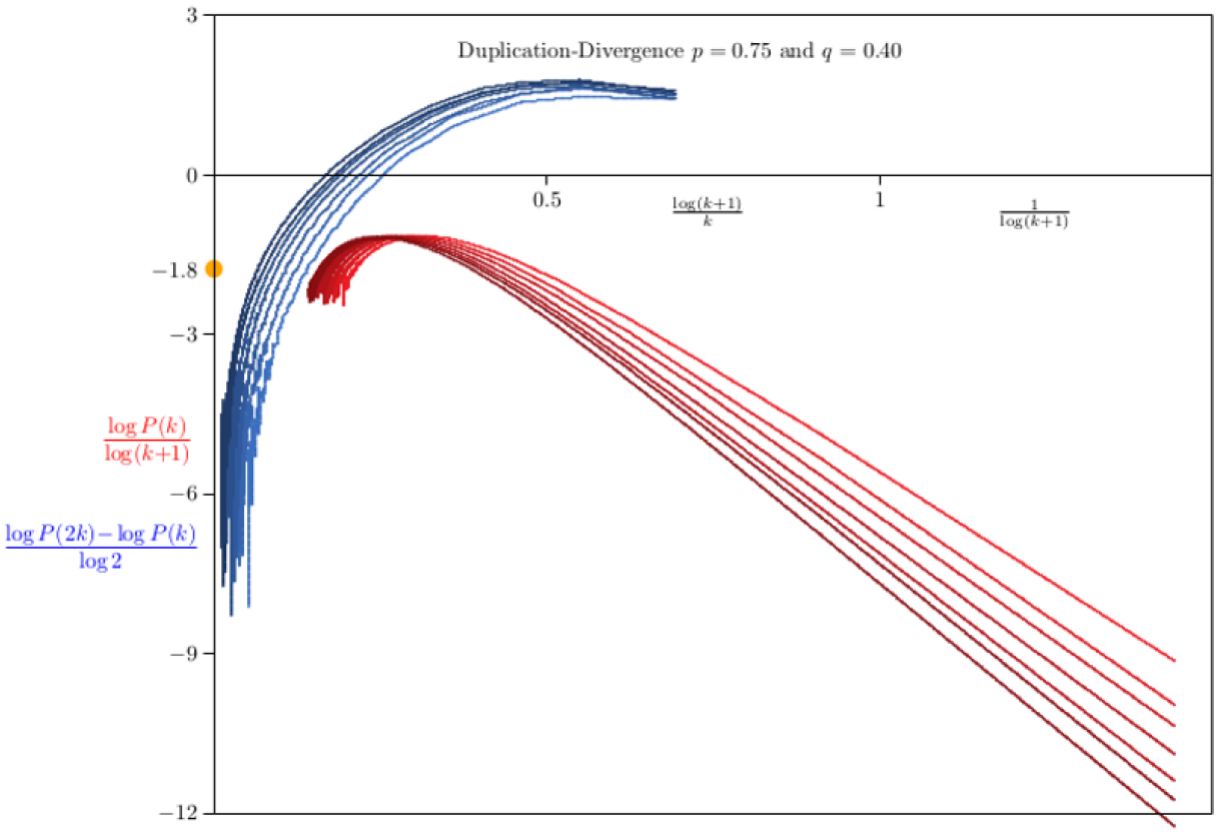
The distribution of degrees in Duplication-Divergence networks with *p* = 0.75 and *q* = 0.40. Data on networks generated by the Duplication-Divergence algorithm. In each case 100 networks were grown and the average degree sequence *P*_*n*_(*k*) computed. The curves on the right are plots of log *P*_*n*_(*k*)/log(*k* + 1) against 1/log(*k* + 1) for *n* ∈ {3125, 6250, 12500, ⋯, 200000}, while those on the left are plots of (log *P*(2*k*) − log *P*(*k*))/log2 as a function of log(*k* + 1)/*k*. The mean field estimate for the exponent *γ* is marked at −*γ* = −1.8 on the left hand axis. The strong correction to scaling evident in these curves makes it difficult to extrapolate to the mean field value for *γ*.

In Fig 8 data for networks grown with *p* = 0.75 and *q* = 0.4 are shown. The curves on the right were obtained by plotting (log *P*(*k*))/log(*k* + 1) averaged over 100 networks of sizes {3125, 6250, 12500, 25000, 50000, 100000, 200000} against 1/log(*k* + 1). The mean field value of *γ* is denoted by the bullet on the left-hand axis. These data show that convergence to this value is very slow—this indicates strong corrections to scaling arising in Eq (42).

An alternative approach is to estimate *γ* by plotting *ζ*(*k*) = (log *P*(2*k*) − log *P*(*k*))/log2 as a function of log(*k* + 1)/*k* (see Eq (4) with *α* = 2). The results are also strongly curved data (left in Fig 8), and while the results are not inconsistent with the mean field value *γ* ≈ 1.9 in this model, however, it seems difficult to extrapolate these curves to a limiting value of *γ*.

**Fig 9:**
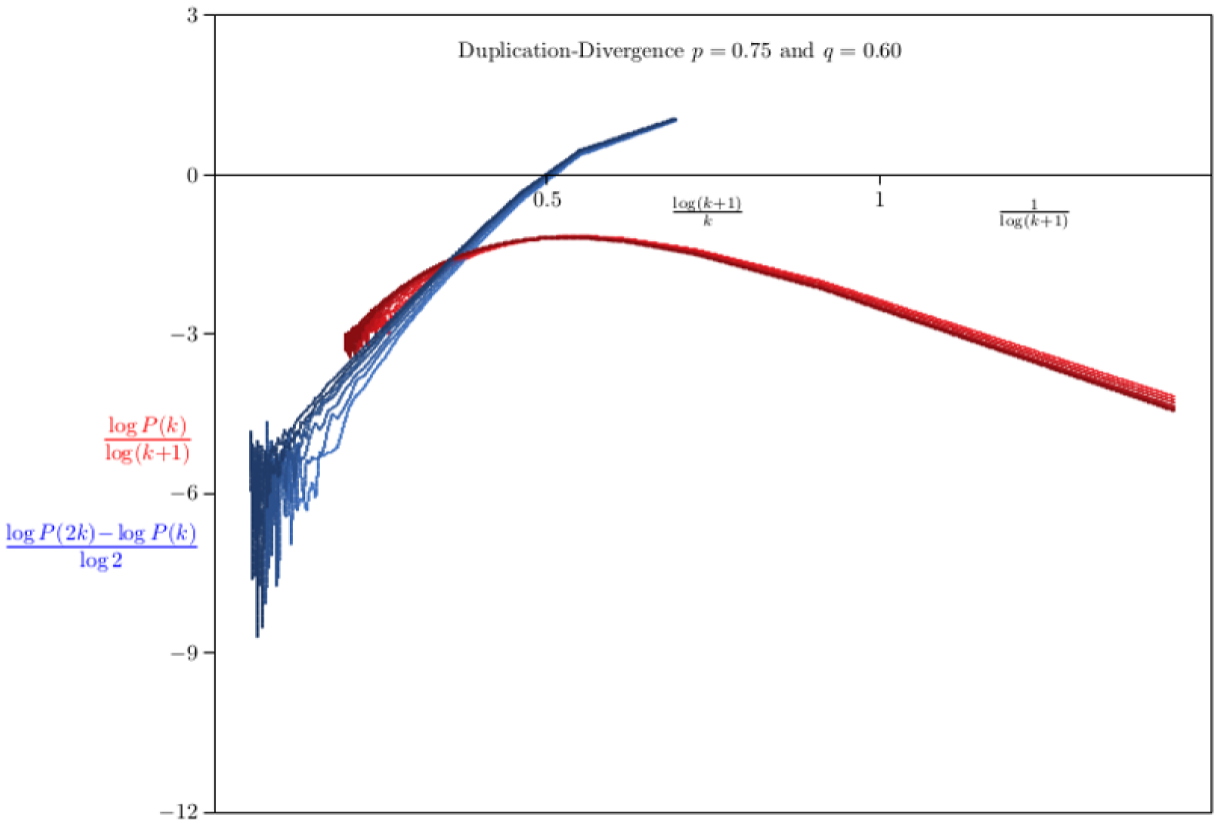
The distribution of degrees in Duplication-Divergence networks with *p* = 0.75 and *q* = 0.60. Data on networks generated by the Duplication-Divergence algorithm. In each case 100 networks were grown and the average degree sequence *P*_*n*_(*k*) computed. The curves on the right are plots of log *P*_*n*_(*k*)/log(*k* + 1) against 1/log(*k* + 1) for *n* ∈ {3125, 6250, 12500, ⋯, 200000}, while those on the left are plots of (log *P*(2*k*) − log *P*(*k*))/log2 as a function of log(*k* + 1)/*k*. Each of these curves can be extrapolated by a quadratic least squares fit to obtained estimates of *γ*. This gives the estimates *γ*_*n*_ for *n* = 3125 × 2^ℓ^ for ℓ = 0, 1, 2, …, 6. Extrapolating the *γ*_*n*_ to *n* = ∞ by a least squares fit *γ*_*n*_ = *γ* + *A*/*n* gives *γ* ≈ 7.4.

If $q=0.60>\frac{1}{2}$ then the results in Fig 9 are seen. The curves of *ζ*(*k*) = (log *P*(2*k*) − log *P*(*k*))/log2 as a function of log(*k* + 1)/*k* have straightened considerably, and each can be extrapolated by a quadratic least squares to obtain an estimate *γ*_*n*_ for each value of *n* = 3125 × 2^ℓ^ (for ℓ = 0, 1, 2, …, 6). This gives estimates {9.68, 8.52, 7.99, 7.95, 7.82, 7.58, 7.05} which can be extrapolated by a least squares fit of *γ*_*n*_ = *γ* + *A*/log *n*, giving the estimate *γ* ≈ 2.87, which is slightly larger than the value predicted by the mean field formula $\gamma =1+\frac{2p}{1+2p-2q}$ (see the paragraph following Eq (46)). This suggests that the approach to limiting behaviour in this model is quite slow, consistent with the remarks after Eq (46) in the previous section.

The average connectivity 〈*k*〉_*n*_ is expected to behave according to Eq (47). In Table 1 〈*k*〉_*n*_ is listed for *p* = 0.75 and *q* = 0.40, *q* = 0.50 and *q* = 0.60. If *q* = 0.4, then Eq (47) suggests that 〈*k*〉_*n*_ ≃ 8.5*n*^0.2^. Computing 〈*k*〉*n* × *n*^−0.2^ from the data in Table 1 gives {5.18, 5.45, 5.65, 5.91, 5.96, 6.01, 6.12}. Plotting these results against 1/log *n* and then linearly extrapolating as *n* → ∞ gives 7.98, close to the value of 8.5 predicted in Eq (47).

**Table 1:** Connectivity data for Duplication-Divergence networks.

*n*	*q* = 0.4	*q* = 0.5	*q* = 0.6
3125	25.9	11.4	5.93
6250	31.3	12.6	6.14
12500	37.3	13.6	6.33
25000	44.8	14.4	6.55
50000	51.9	15.5	6.64
100000	60.1	16.8	6.75
200000	70.3	17.7	6.88

If *q* = 0.5, then Eq (47) suggests that 〈*k*〉_*n*_ ≃ 1.5log *n* since *p* = 0.75. Dividing the results in Table 1 by log *n* for each value of *n* gives the results {1.42, 1.44, 1.44, 1.42, 1.43, 1.46, 1, 45}. The average of this is close to the predicted value of 1.5.

Finally, if *q* = 0.6 then the data appear to approach a constant. Extrapolating these results using the model *A* + *B*/log(*n*) gives the estimated limiting value 8.72. By Eq (5) this indicates that *γ* = 2.13, a value which is quite close to 2.15, the value predicted by the formula $\gamma =1+\frac{2p}{1+2p-2q}$ in the paragraph following Eq (46).

### Solé evolutionary networks

The Solé model [12, 16] modifies Duplication-Divergence model by using duplication and network rewiring as the basic elementary moves. As before, the duplication of nodes is an implementation of gene duplication, and the network rewiring is based on the loss and gain of protein interactions in the bulk of the network [4]. Thus, the algorithm grows networks based on a model of gene duplication and the rewiring of protein interactions; both these processes drive the evolution of the interactome.

The elementary move of the algorithm is as follows: A node in the network is chosen uniformly and randomly, and duplicated to form a progenitor-progeny pair. The progeny will have the same interactions as the progenitor. This network is updated in the rewiring step which has two parts: Bonds incident with the progeny protein are deleted with probability *δ*, and new bonds are added in the network between nodes (excluding the progenitor protein) are created with probability *α*. This implementation differs in two ways from the Duplication-Divergence algorithm. In the Solé model there are no self-interacting nodes, and the formation of new bonds in the rewiring steps only occurs in the Solé model.

**Fig 10:**
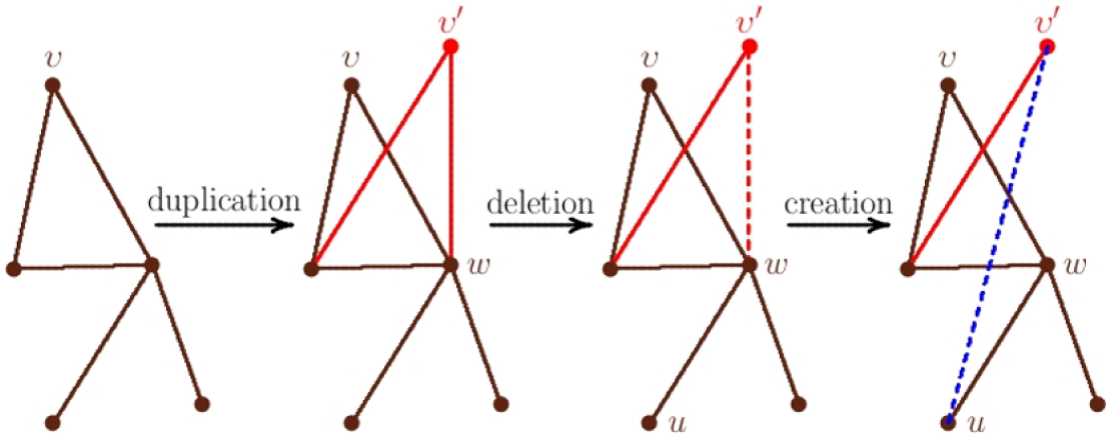
The Solé evolutionary algorithm. The duplication-deletion-creation iterations of the Solé algorithm. A site is duplicated, some bonds incident on it are deleted with probability *δ* and new bonds incident on it are created with probability *α*.

The basic iterative step of the Solé algorithm is shown in Fig 10.

A Solé evolutionary network of order *N* nodes is grown as follows:

**Solé evolutionary algorithm:**

1. Initiate the network with one node *x*_0_ and apply the following steps iteratively;
2. Choose a node *υ* uniformly and duplicate it to a new node *υ*′;
3. For each bond 〈*w* ∼ *υ*〉 incident with the chosen node *υ*, add the bond 〈*w* ∼ *υ*′〉 incident with the duplicated node *υ*′;
4. Delete each bond 〈*w* ∼ *υ*′〉 added in step 3 with probability *δ* independently;
5. For all nodes *u* not adjacent to the chosen node *υ*, create the bond 〈*u* ∼ *υ*′〉 with probability *α*;
6. Stop the algorithm when a network of order *N* is grown.

The algorithm has two parameters (*δ*, *α*). If *δ* = 0 and *α* = 1 then the algorithm grows complete simple networks. More generally, if *α* > 0 then on average roughly *αN* bonds are added to a network of order *N*. This shows that the algorithm grows networks of size *O*(*N*^2^)—that is, Solé networks are rich in bonds.

#### Mean field theory for Solé networks

Let *E*_*n*_ be the total number of bonds in a Solé network after *n* iterations of the algorithm, and let 〈*k*〉_*n*_ be the connectivity of the network (that is, the average degree of nodes) after *n* iterations (so that 2*E*_*n*_ = *n*〈*k*〉_*n*_). In the mean field approximation the node in step 2 of the algorithm has degree 〈*k*〉_*n*_ and this number of bonds is added in step 3, while, in a similar way, *δ*〈*k*〉_*n*_ bonds are removed in step 4. In step 5 there are *n* − 〈*k*〉_*n*_ choices in the mean field for the node *u* not adjacent to *υ*′ and each bond 〈*u* ∼ *υ*′〉 is added with probability *α*. This shows that the number of bonds after *n* + 1 iterations is given by the recurrance relation

$$\begin{array}{c} E_{n+1}=E_{n}+\left(1-\delta \right){\left\langle k\right\rangle }_{n}+\alpha \left(n-{\left\langle k\right\rangle }_{n}\right). \end{array}$$ (49)

Since 2*E*_*n*_ = *n*〈*k*〉_*n*_ this becomes

$$\begin{array}{c} E_{n+1}-E_{n}=\alpha n+\frac{2}{n}\left(1-\delta -\alpha \right)\;E_{n}, \end{array}$$ (50)

which is a mean field recurrence relation for *E*_*n*_.

Taking *n* → *t*, a continuous time variable, and approximating *E*_*n*_ by *E*_*t*_, and approximating the finite difference as a derivative, gives the following differential equation for *E*_*n*_:

$$\begin{array}{c} \frac{d}{dt}E_{t}=\alpha t+\frac{2}{t}\left(1-\alpha -\delta \right)\;E_{t}. \end{array}$$ (51)

Solving this equation and letting *t* → *n* again gives the approximate mean field solution for *E*_*n*_:

$$\begin{array}{c} E_{n}\approx \frac{\alpha n^{2}}{2(\alpha +\delta )}+\frac{\left(\alpha +2\delta \right)n^{2(1-\alpha -\delta )}}{2(\alpha +\delta )}. \end{array}$$ (52)

Eq (52) shows that the number of bonds is proportional to *n*^2^, so that networks created by this algorithm are dense, except when *α* = 0. Comparison to Eq (7) suggests that *γ* ≤ 1 in this model. Notice that there is no logarithmic factor in the denominator, and that *E*_*n*_ = Θ(*n*^2^). This is consistent with a mean field value *γ* < 1 (and this requires that *P*_*n*_(*k*) be modified so that it is a normalisable probability distribution). With these results, it is reasonable to expect that, in the mean field,

$$\begin{array}{c} \gamma \leq 1. \end{array}$$ (53)

If *α* = 0 then Eq (52) gives *E*_*n*_ ∼ *n*^2−2*δ*^ and comparison to Eq (7) gives

$$\begin{array}{c} \gamma =1+2\delta ,\;\text{if}\;\alpha =0. \end{array}$$ (54)

#### Numerical results for Solé networks

Similar to Barabási-Albert and Duplication-Divergence networks, Solé networks can be grown numerically by implementing the algorithm as given above, using sparse matrix routines to efficiently store the adjacency matrix of the network. The larger size of networks makes these more difficult to grow, and our algorithms sampled efficiently to networks of size 51200 bonds.

**Fig 11:**
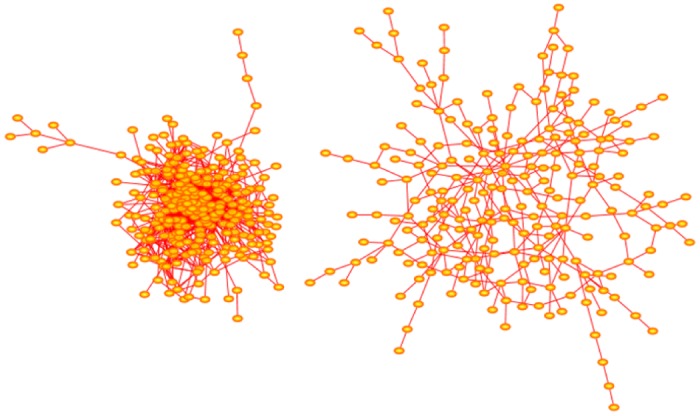
Solé evolutionary networks. The network on the left was generated with *δ* = 0.25 and *α* = 0.005. Its has order 279 and has 47 nodes with degrees exceeding $\sqrt{279}$ and so qualify as hubs. The largest few of these hubs have degrees {40, 41, 62, 80}. This algorithm creates dense networks as seen here, even for small values of *α*. Increasing the value of *δ* gives more extended networks. The network on the right was generated with *δ* = 0.75 and *α* = 0.005 and grown to order 230. None of its nodes qualify as hubs. The arrangement of nodes and bonds in these networks was created using the prefuse force directed lay-out in Cytoscape 3.4.0 [17].

Solé networks are rich in bonds. This is seen, for example, in Eq (52), which shows that *E*_*n*_ ∝ *n*^2^ if *α* > 0. In Fig 11 two examples of networks generated by the Solé algorithm are shown. If *δ* < 0.5, then the networks have a dense appearance dominated by a few hubs. If *δ* > 0.5, then the networks appear more extended, often with no nodes qualifying as hubs under the definition that the degree of a hub in a network of order *n* is at least $\left\lfloor \sqrt{n}\right\rfloor$. The networks in Fig 11 were generated with *α* = 0.005, and increasing the value of *α* quickly increases the number of bonds.

The mean field result that *γ* ≤ 1 has implications for the scaling of Solé networks. In particular, *P*_*N*_(*k*) in Eq (1) is not normalisable for infinite networks if *γ* ≤ 1 and so is not a valid candidate degree distribution in this model. The degree distribution can be modified to

$$\begin{array}{c} P\left(k\right)≃C_{o}\;k^{-\gamma }D\left(n^{-\phi }k\right) \end{array}$$ (55)

where *D*(*x*) is a function of the combined (or scaled) variable *x* = *n*^−*ϕ*^ *k*. That is, as *n* → ∞, *k* is rescaled by *n*^−*ϕ*^ and *k*^*γ*^ *P*(*k*) approaches a limiting distribution proportional to *D*(*x*).

**Fig 12:**
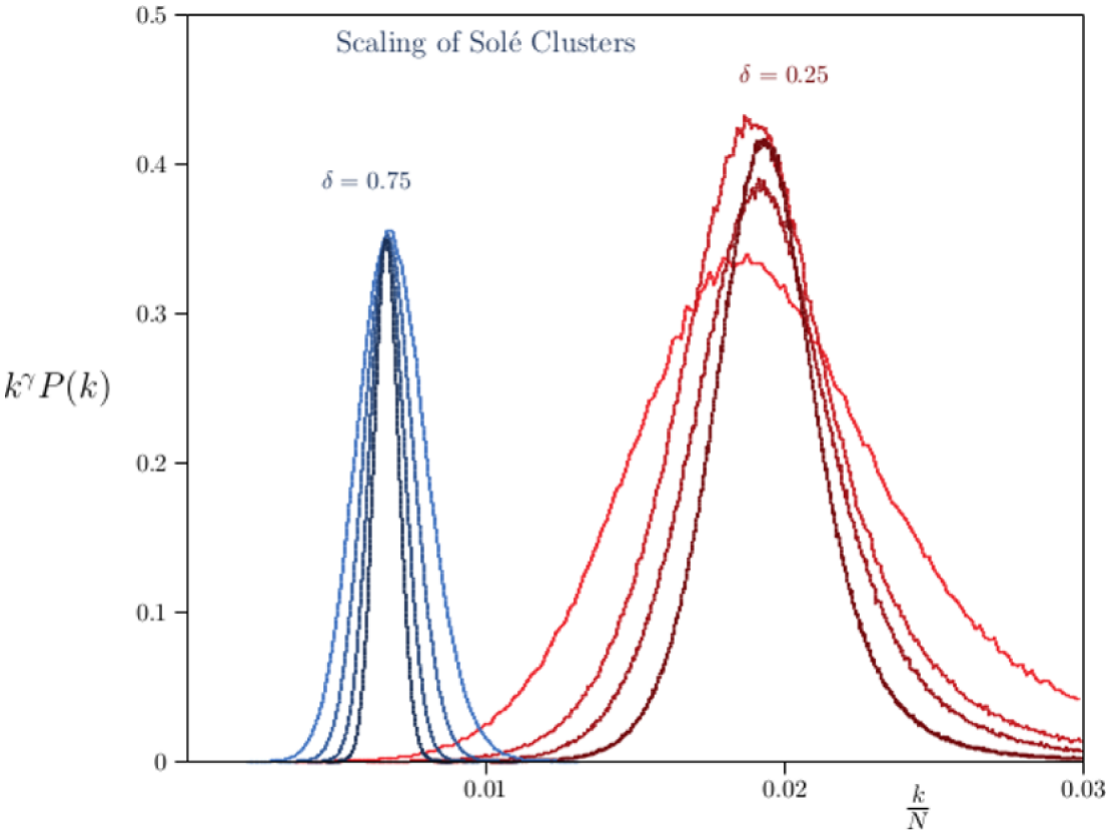
Scaling of Solé evolutionary networks. Plotting *k*^*γ*^ *P*_*N*_(*k*) against *N*^−*ϕ*^ *k* for networks generated by the Solé Evolutionary algorithm gives the distributions above. On the left the results are shown for networks grown with *δ* = 0.75 and *α* = 0.005. The choices *γ* = 1/2 and *ϕ* = 1 uncovers a distribution as shown where the order of the networks are *N* = 100 × 2^*n*^ for *n* = 6, 7, 8, 9. A similar distribution, but with *γ* = 2/3 and *ϕ* = 1, is seen when networks are grown with *δ* = 0.25 and *α* = 0.005. It is not known that the value of *γ* changes discontinuously as *δ* increases from 0.25 to 0.75.

This can be tested numerically by plotting *n*^*γ*^ *P*(*k*) as a function of *x* = *n*^−*ϕ*^ *k*. For the proper choices of *γ* and *ϕ* it is expected that *n*^*γ*^*P*(*k*) ≃ *C*_*o*_*D*(*x*) for a wide range of values of *n* (that is, the data should approach a limiting curve as *n* → ∞). The result is shown in Fig 12 for (*δ* = 0.25, *α* = 0.005) and (*δ* = 0.75, *α* = 0.005). These are plots on the same graph for *n* = 100 × 2^*n*^ for *n* ∈ {6, 7, 8, 9} (other curves at smaller values of *N* are left away to give a clearer picture).

The data for *δ* = 0.75 are the cluster of peaks to the left, rescaled by choosing *ϕ* = 1 and $\gamma =\frac{1}{2}$, while the cluster of peaks to the right is for *δ* = 0.25 with *ϕ* = 1 and $\gamma =\frac{2}{3}$. With increasing *n* the data appear to approach a single underlying curve if $\gamma =\frac{1}{2}$ in the one instance, and $\gamma =\frac{2}{3}$ in the other instance. Both these values are consistent with the mean field expectation that *γ* ≤ 1 in this model. Further refinements in this scaling assumption may be necessary, since the curves are still becoming narrower with increasing *n*. It is not clear that these approach a limiting curve as *n* → ∞, although the data for *δ* = 0.75 suggest this to be the case. In these cases the curves are sharply peaked with a mean of about 0.02 if *δ* = 0.25 and about 0.007 if *δ* = 0.75.

Since the curve *D*(*x*) is sharply peaked at a constant value *c*_*o*_ of the rescaled variable *x*, the connectivity of Solé networks is estimated by treating *D*(*x*) as concentrated at *c*_*o*_ and then (assuming that *ϕ* = 1 and approximating the connectivity)

$$\begin{array}{c} {\left\langle k\right\rangle }_{n}∼\frac{\int_{0}^{\infty }k^{1-\gamma }D\left(k/n^{\phi }\right)\;dk}{\int_{0}^{\infty }k^{-\gamma }D\left(k/n^{\phi }\right)\;dk}∼\frac{{\left(n^{\phi }\right)}^{2-\gamma }}{{\left(n^{\phi }\right)}^{1-\gamma }}∼n^{\phi }. \end{array}$$ (56)

In other words, the connectivity of Solé networks should increase linearly with *n*^*ϕ*^ (and since *ϕ* = 1, linearly with *n*). In Table 2 the connectivities of Solé networks for *δ* = 0.25 and *δ* = 0.75 (with *α* = 0.005) are listed. Non-linear least squares fits to the data show that *ϕ* = 1.01 when *δ* = 0.25 and *ϕ* = 0.99 when *δ* = 0.75. That is, these results are consistent with the value *ϕ* = 1 seen above.

**Table 2:** Connectivity data for Solé networks.

*n*	*δ* = 0.25	*δ* = 0.75
100	2.95	1.50
200	4.46	1.94
400	7.59	2.94
800	14.75	5.36
1600	30.46	10.64
3200	59.94	21.26
6400	122.78	45.57
12800	245.35	85.18
25600	496.87	170.35
51200	994.54	340.76

### The iSite model of network evolution

Protein interaction networks evolve by mutations in proteins which change the interactions of the proteins in the network. In the Duplication-Divergence algorithm, a mutated protein loses its interactions randomly. This random deletion of interactions is a good first order approximation to the evolution of networks. The iSite model refines this by giving structure to nodes in the network by introducing *iSites* on nodes as localities of the interaction sites on a protein [13, 14]. Subfunctionalization of interaction sites in the iSite model is implemented by silencing iSites, and adding interactions with reduced probability if the iSite is not silenced.

The implementation of the iSite algorithm relies in the first place on duplication of nodes, and then subfunctionalization of iSites on the nodes. The subfunctionalization of iSites is implemented by randomly deleting of bonds incident to duplicated iSites, *and* by the silencing of iSites by turning them off. These processes are models of random mutations which cause the loss of information in the genome (and leave behind non-coding remnants of genes). A process of spontaneously creating new iSites is not in the iSites algorithm, although this is a possible refinement which may be introduced in the algorithm.

**Fig 13:**
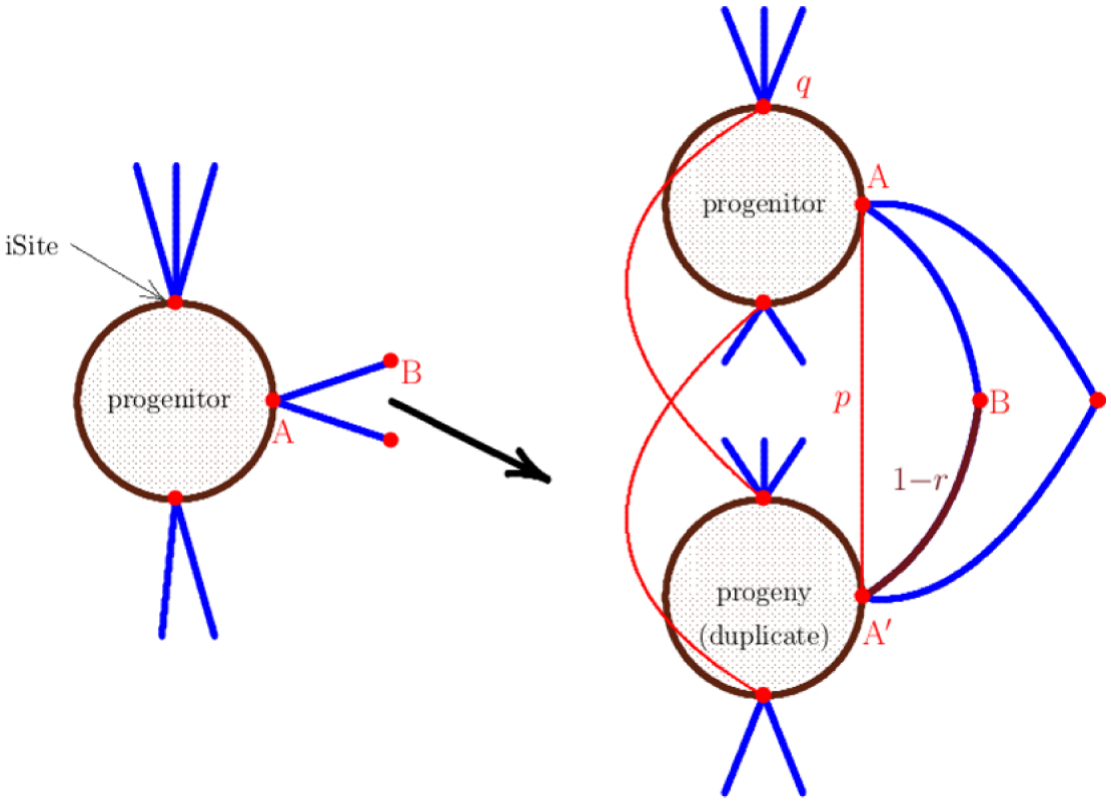
The iSite evolutionary algorithm. The duplication-deletion iterations of the iSite algorithm. A node together with its iSites is duplicated, and some bonds incident with the duplicated iSites are deleted with probability *r*. New bonds between a self-interacting iSite and its duplicate are inserted with probability *p*, and iSites are silenced with probability *q*.

The elementary move of the iSite algorithm is illustrated schematically in Fig 13. A uniformly chosen node is duplicated into a progenitor-progeny pair (and so also duplicating the iSites of the progenitor onto the progeny). If the duplicated iSite is self-interacting, then bonds are added between the iSite on the progenitor and the duplicated iSite on the progeny with probability *p*—this allows for subfunctionalization of the duplicate iSites. Bonds incident with the iSites on the progenitor are duplicated with reduced probability *r*, and iSites on the progenitor or progeny nodes are silenced with probability *q*. If an iSite is silenced, then all bonds incident with it are deleted. Notice that subfunctionalization enters in several ways, both in the duplication of self-interacting iSites, in the duplication of bonds, and in the silencing of iSites.

The algorithm is implemented as follows:

**iSite evolutionary algorithm:**

1. Initiate the network with one node *x*_0_ with *I* active iSites (each of which is self-interacting with probability *p*) and iterate the following steps;
2. Choose a progenitor protein *υ* uniformly in the network and duplicate it, and its associated iSites *A*, to a successor protein *υ*′ with duplicated iSites *A*′;
  (a) A duplicated iSite *A*′ ∈ *υ*′ is active with probability *q* if it is duplicated from an active iSite on *A* ∈ *υ*, and silenced otherwise;
  (b) An active duplicated iSite *A*′ ∈ *υ*′ is self-interacting with probability *p* if it is duplicated from a self-interacting iSite on *A* ∈ *υ*, and not self-interacting otherwise;
  (c) If a silenced iSite *A* is duplicated to iSite *A*′, then *A*′ is also silenced;
3. Add bonds as follows:
  (a) If iSite *A* ∈ *υ* is self-interacting and *A* is duplicated to iSite *A*′ ∈ *υ*′, then add the bond 〈*A* ∼ *A*′〉 if *A*′ is not silenced;
  (b) If 〈*A* ∼ *B*〉 is a bond incident with iSite *A* on the progenitor *υ*, and *A* is duplicated to iSite *A*′ on the duplicate *υ*′, then 〈*A* ∼ *B*〉 is duplicated to 〈*A*′ ∼ *B*〉 with probability 1 − *r* provided that *A*′ is not silenced;
4. Iterate the algorithm from step (2) and stop the iterations when a network of order *N* is grown.

#### Mean field theory for the iSite model

Let nodes in the network correspond to proteins, and let *i*_*j*_(*n*) be the number of active iSites on node *j* after *n* iterations of the algorithm. Denote the degree of node *j* by *k*_*j*_(*n*) (that is the total number of bonds with one end-point in node *j*), and let *E*_*n*_ be the number of bonds of the network (this is the *size* of the network). Then 2*E*_*n*_ = ∑_*j*_ *k*_*j*_(*n*).

The average number of active iSites per node is $i\left(n\right)=\frac{1}{n}\sum_{j}i_{j}\left(n\right)$. With each iteration *i*(*n*) iSites are created, of which *qi*(*n*) are silenced, in the mean field. This gives the following recurrance relation for *i*(*n*):

$$\begin{array}{c} (n+1)\;i(n+1)=n\;i(n)+(1-q)\;i(n). \end{array}$$ (57)

The exact solution of this recurrance is

$$\begin{array}{c} i\left(n\right)=\frac{i(0)\;\Gamma (1-q+n)}{n!\;\Gamma (1-q)} \end{array}$$ (58)

where Γ is the gamma function with the property that Γ(*x* + 1) = *xΓ*(*x*) and Γ(1) = 1. Notice that *i*(0) = *I*, where *I* is the number of iSites on the source node *x*_0_.

For large *n* the Γ-function and the factorial have well known asymptotics (namely the Stirling approximation [27]), so that

$$\begin{array}{c} i\left(n\right)≃\frac{I\;n^{-q}}{\Gamma (1-q)}. \end{array}$$ (59)

This shows that with increasing *n* the total number of iSites grows proportionally to *n*^1−*q*^. If *q* = 0, then this is linear in *n* since no iSites become silenced, and if *q* = 1, then the number approaches a constant.

The total number of bonds in the network increases after *n* iterations by the recurrance

$$\begin{array}{c} E_{n+1}=E_{n}+\frac{2(1-r)}{n}\;E_{n}+p\;i\left(n\right), \end{array}$$ (60)

since there are on average $\frac{2}{n}E_{n}$ bonds incident to each node, and the probability that each one of them is duplicated is 1 − *r*, and there are on average *i*(*n*) iSites per node, and the probability that each of these is self-interacting is *p*.

Using the asymptotic solution for *i*(*n*) and approximating this recurrence by a differential equation gives

$$\begin{array}{c} \frac{d}{dt}\;E_{t}=\frac{2(1-r)}{t}\;E_{t}+\frac{pI}{\Gamma (1-q)}\;t^{-q}. \end{array}$$ (61)

This equation can be solved, and using the initial condition *E*_1_ = 0, the result is

$$\begin{array}{c} E_{t}=\frac{pI}{\left(1+q-2r)\;\Gamma (1-q\right)}\left(t^{2-2r}-t^{1-q}\right). \end{array}$$ (62)

Thus, the average degree of a node is equal to $\frac{2}{n}E_{n}$, so that the connectivity of iSite evolutionary networks is given by

$$\begin{array}{c} {\left\langle k\right\rangle }_{n}≃\frac{2pI}{\left(1+q-2r)\;\Gamma (1-q\right)}\left(t^{1-2r}-t^{-q}\right) \end{array}$$ (63)

in the mean field. This shows that the large *n* value of 〈*k*〉_*n*_ is dominated by the larger of −*q* and 1 − 2*r*. In particular, if $r<\frac{1}{2}\left(1+q\right)$, then 〈*k*〉_*n*_ ∼ *n*^1−2*r*^.

By Eq (7) one may determine *γ* for this model:

$$\begin{array}{c} \gamma =\left\{ \begin{array}{cc} 1+2r, & \text{if}\;r<\frac{1}{2}\left(1+q\right);\\ 2+q, & \text{if}\;r>\frac{1}{2}\left(1+q\right). \end{array}\right. \end{array}$$ (64)

If 2*r* = 1 + *q*, then a different solution is obtained, namely

$$\begin{array}{c} E_{t}=\frac{pI}{\Gamma (1-q)}\;t^{1-q}\log t. \end{array}$$ (65)

This shows that *γ* = 2 + *q* in this case as well, but there is also a logarithmic correction to the growth of *E*(*t*), and so there is a logarithmic factor in the expression for 〈*k*〉_*n*_.

#### Modified iSite evolutionary algorithm

The subfunctionalization of proteins can be refined by introducing in the iSite algorithm the probability of creating new iSites on the progeny node with a probability *s*. This changes the algorithm as follows.

**Modified iSite evolutionary algorithm:**

Implement the algorithm as above but introduce the parameter *s* and create new active iSites by replacing step 2 in the iSite evolutionary algorithm by

2 Choose a progenitor node *υ* uniformly in the network and duplicate it, and its associated iSites *A*, to a progeny node *υ*′ with duplicated iSites *A*′;
  (a) A duplicated iSite *A*′ ∈ *υ*′ is active with probability *q* if it is duplicated from an active iSite on *A* ∈ *υ*, and silenced otherwise;
  (b) An active duplicated iSite *A*′ ∈ *υ*′ is self-interacting with probability *p* if it is duplicated from a self-interacting iSite on *A* ∈ *υ*, and not self-interacting otherwise;
  (c) If a silenced iSite *A* is duplicated to iSite *A*′, then *A*′ is also silenced;
  (d) With probability *s* create an active iSite *C* on the progeny node *υ*′, where *C* is self-interacting with probability *p*.

The recurrence for the average number of active iSites per node *i*(*n*) (see Eq (58)) is modified to

$$\begin{array}{c} \left(n+1)\;i(n+1)=n\;i(n)+(1+s-q)\;i(n\right) \end{array}$$ (66)

in the Modified iSite evolutionary algorithm. The exact solution is obtained by replacing *q* by *q* − *s* in Eq (58), and the asymptotic approximation of the solution is given by

$$\begin{array}{c} i\left(n\right)≃\frac{I\;n^{s-q}}{\Gamma (1+s-q)}, \end{array}$$ (67)

as seen in Eq (59).

The total number of bonds in the network, *E*_*n*_, still satisfies Eq (60), and so it follows from Eqs (62)–(64), that for the modified iSite evolutionary algorithm (notice the condition that *q* < *r* + *s*):

$$\begin{array}{c} E_{n}=\frac{pI}{\left(1+q-s-2r)\;\Gamma (1+s-q\right)}\left(n^{2-2r}-n^{1+s-q}\right). \end{array}$$ (68)

This shows that the connectivity of Modified iSite networks is given by

$$\begin{array}{c} {\left\langle k\right\rangle }_{n}≃\frac{2pI}{\left(1+q-s-2r)\;\Gamma (1+s-q\right)}\left(n^{1-2r}-n^{s-q}\right). \end{array}$$ (69)

The value of the scaling exponent is seen from above to be given by

$$\begin{array}{c} \gamma =\left\{ \begin{array}{cc} 1+2r, & \text{if}\;r<\frac{1}{2}\left(1+q-s\right);\\ 2+q-s, & \text{if}\;r>\frac{1}{2}\left(1+q-s\right). \end{array}\right. \end{array}$$ (70)

with a correction factor in the expression for 〈*k*〉_*n*_ if 2*r* = (1 + *q* − *s*).

#### Numerical results for iSite networks

**Fig 14:**
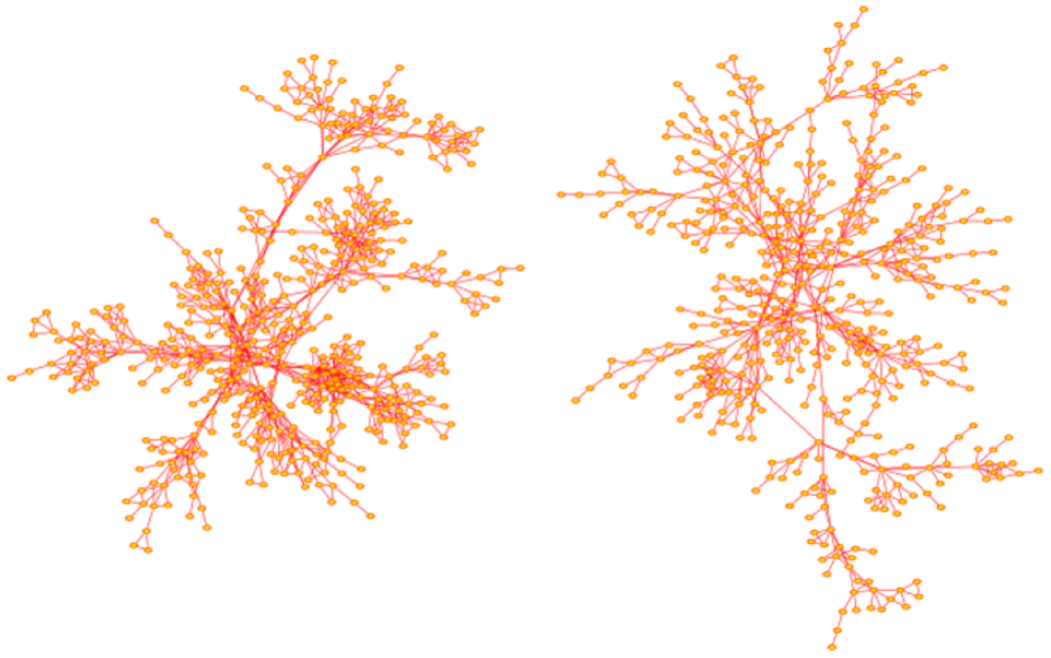
iSite evolutionary networks. The network on the left was generated with 4 iSites per node, *p* = 0.5, *q* = 0.1 and *r* = 0.8, and the network on the right was generated with 2 iSites per node, and with *p* = 0.5, *q* = 0.1 and *r* = 0.8. The order of the network on the left is 501 and on the right, 491. The network on the left has two nodes qualifying as hubs, of degrees {23, 25}, while the network on the right has none. The arrangement of nodes and bonds in these networks was created using the prefuse force directed lay-out in Cytoscape 3.4.0 [17].

The iSite algorithm was coded and networks were grown to compute averaged statistics. Examples of iSite networks generated by the algorithm are shown in Fig 14. The algorithm was then used to sample networks of size up to 200,000.

The connectivity 〈*k*〉_*n*_ of iSite networks for *I* = 3 iSites per node, and with *p* = 0.5, *q* = 0.4 and *r* = 0.3, is shown in Table 3. By Eq (5), $\log\langle k\rangle ≃\log\frac{\gamma -1}{2-\gamma }+\left(2-\gamma \right)\log n$. Least squares fit to the data in Column 2 gives $\log\frac{\gamma -1}{2-\gamma }\approx 1.0211$, and (2 − *γ*) = 0.258. Solving for *γ* gives in the first instance *γ* = 1.735 and in the second *γ* = 1.742. Since 2*r* < 1 + *q* in this case, the mean field value of *γ* is *γ* = 1 + 2*r* = 1.6, close to these estimated values.

**Table 3:** Connectivity data for iSite networks.

***n***	**Column 1**	**Column 2**	**Column 3**	**Column 4**
3125	22.385	20.701	4.756	6.648
6250	26.524	25.752	4.770	6.556
12500	31.395	29.137	4.677	6.579
25000	37.808	35.308	4.733	6.358
50000	45.931	42.244	4.579	6.299
100000	54.830	50.035	4.584	6.204
200000	64.668	59.284	4.649	6.071
**Columns**	***I***	***p***	***q***	***r***
Column 1:	*I* = 3	*p* = 0.5	*q* = 0.4	*r* = 0.3
Column 2:	*I* = 5	*p* = 0.5	*q* = 0.4	*r* = 0.3
Column 3:	*I* = 3	*p* = 0.5	*q* = 0.05	*r* = 0.8
Column 4:	*I* = 5	*p* = 0.5	*q* = 0.05	*r* = 0.8

**Fig 15:**
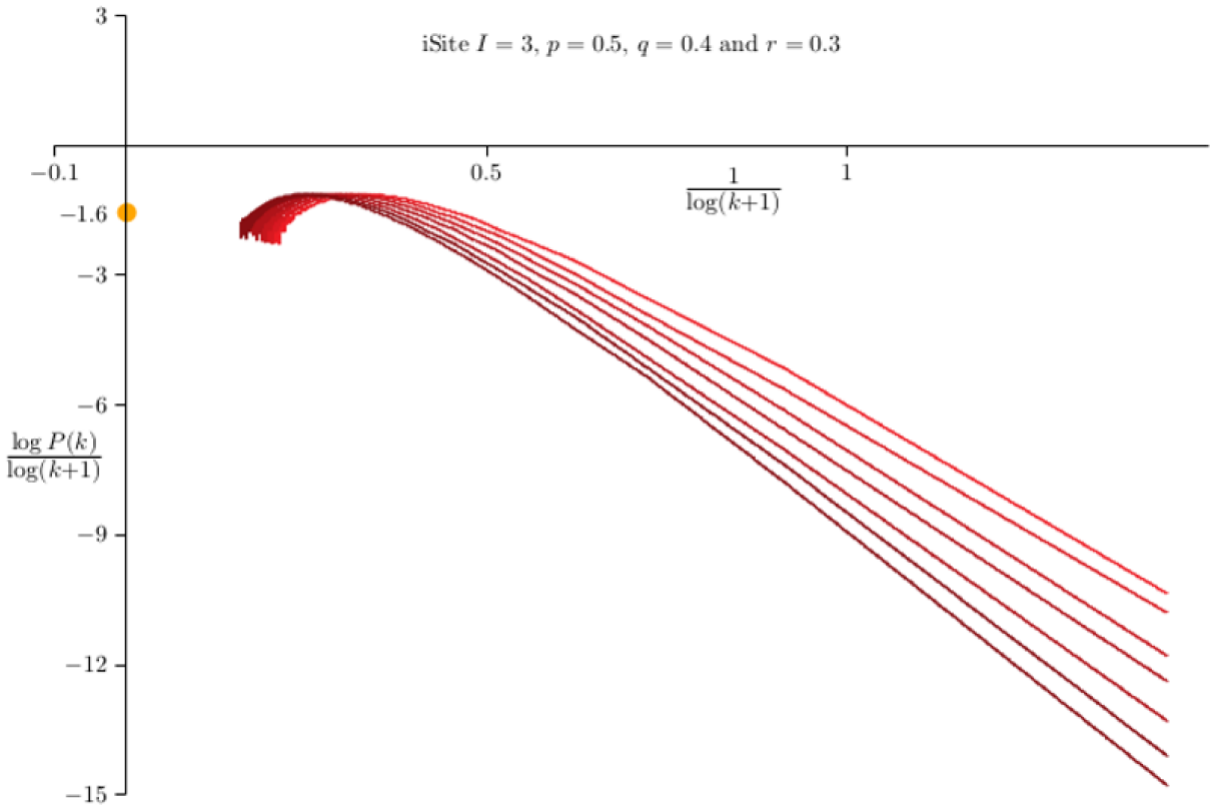
iSite evolutionary networks with *I* = 3, *p* = 0.5, *q* = 0.4 and *r* = 0.3. Data on networks generated by the iSite evolutionary algorithm. In each case 500 networks were grown and the average degree sequence *P*_*n*_(*k*) computed. The curves are plots of log *P*_*n*_(*k*)/log(*k* + 1) against 1/log(*k* + 1) for *n* ∈ {3125, 6250, 12500, ⋯, 200000}. As *k* → ∞, then the curves are expected to pass through −*γ* on the *y*-axis, and its mean field value is *γ* = 1 + 2*r* = 1.6—this value is marked on the *y*-axis.

Data for *I* = 5 and with the same values of (*p*, *q*, *r*) = (0.5, 0.4, 0.3) are shown in Table 3 as well (see Fig 15). Changing the value of *I* (the number of iSites per node) should not change the value of *γ*, and this appears to be the case here. A least squares fit to the data in Column 3 and determining *γ* as above gives *γ* = 1.737 and *γ* = 0.7498, very close to the values above.

If *p* = 0.5, *q* = 0.05 and *r* = 0.8, then 2*r* > 1 + *q*, and in this case *γ* = 2 + *q*. If the number of iSites per node is *I* = 3, then the data in Table 3 gives a constant value for 〈*k*〉, and for *I* = 5 a slightly decreasing numerical estimate. The mean field value of *γ* in these cases is 2.05, and a least squares fit gives *γ* ≈ 2.009 if *I* = 3 and *γ* ≈ 2.022 if *I* = 5 (where the coefficient of log *n* in the least squares fit is 2 − *γ*). These results are consistent with the mean field results obtained above, since it shows that the value of *γ* is close to 2 + *q*.

## Conclusions

In this paper a number of algorithms used for generating networks in molecular biology were examined. Mean field theory for the algorithms was in some cases reviewed, and in other cases newly presented, and also refined. The algorithms include the Barabási-Albert [6], Duplication-Divergence [10], Solé [12] and iSite algorithms [13, 14], and these were in some cases modified by the introduction of more general elementary moves. The modified and variant algorithms in this paper are new, and the mean field analyses of the Barabási-Albert and Duplication-Divergence algorithms are reviews of analyses done in references [9, 11]. The analyses of the variants and modified versions of the algorithms, and the analyses of the Solé and iSite algorithms are new.

The mean field result for the *γ*-exponent for the modified Barabási-Albert algorithm in Eq (19) is new, and it reduces to the known mean field value of *γ* for the Barabási-Albert algorithm when *λ* = 1 and *A* = 0 (see reference [9]). Similarly, the mean field estimate for the Duplication-Divergence algorithm in Eq (46) generalises a result in references [10, 11] and gives generally good estimates of *γ*. However, as noted below Eq (46), a refined estimate for *γ* gives values which are too small and the value of *γ* in the regime that 2*q* < 1 + 2*p* remains an open question.

The mean field estimates and bounds in Eqs (53) and (54) for the Solé algorithm, and in Eqs (64) and (70) for the iSite algorithm, and its modified version, are new. The mean field values for *γ* for the Solé algorithm give results which are well-supported by the data collected in Fig 12. This is similarly the case for the iSite algorithm, and for its modified version, where values of *γ* consistent with mean field predictions are obtained from our numerical simulations.

The efficient implementation of these algorithms was also examined, and sparse matrix routines (or, more general, hash-coding; see for example reference [28]) were used to optimize the implementation. This gives computer algorithms which can generate very large networks efficiently, and networks of order 200,000 nodes were routinely sampled. We also explored even larger networks, up to order 3 million, but did not use those in our data analysis.

The adjacency matrix of a network of size *E* bonds can be stored (using sparse matrix routines) in an array of size *O*(*E*). This means that the implementation of these network growth algorithms has average case space complexity *O*(*E*).

Hash coding allows for the efficient implementation of routines which search, insert or delete entries in arrays storing the networks. These routines have average time complexity *O*(1) [29], (and worst case time complexity *O*(*E*) for searches, inserting and deleting bonds, due to collisions if a hash table is densely populated).

Generally, the time complexity of algorithms should grow as *O*(*E*^*τ*^) if networks of size *E* are grown (where *τ* is an exponent dependent on the particular algorithm). For example, networks of size *E* bonds can be generated using *O*(*E*) computer memory, and the Duplication-Divergence and iSite algorithms can be implemented with *O*(*n*^*τ*^) time complexity to grow networks of order *n* nodes (and where *n* ≤ *E*). An examination of these algorithms (the Duplication-Divergence and iSite algorithms) suggests that an optimal implementation will have *τ* ≈ 1 (if the size of the hash tables is much larger than *n*).

The Barabási-Albert and Solé algorithms (with their modified and variant implementations) should have average time complexity of *O*(*n*^2^) for growing networks of order *n* nodes. This follows because each iteration of the algorithms has to explore all nodes in the current network for the possible insertion of new bonds.

Data on the time complexity of the algorithms are shown in Table 4. The data displayed are the average time *T* to grow one network of order *n*. Assuming that *T* = *C*_0_ *n*^*τ*^ and fitting log *T* to log *n*, least squares estimates of *τ* can be obtained. For example, it is expected that *τ* = 2 for the Barabási-Albert algorithm, while the estimate obtained in the table is *τ* ≈ 1.97. This is consistent with the expectation that the time complexity of the algorithm is *O*(*n*^2^) in an optimal implementation. This is similarly seen for the modified and variant implementation of the Barabási-Albert algorithm, and for the Solé algorithm.

**Table 4:** Computational Time Complexity of Implemented Algorithms.

Algorithm	*n* = 6250	*n* = 12500	*n* = 25000	*n* = 50000	*τ*
Bar-Alb (*p* = 0)	0.602	2.51	9.03	38.0	1.97
Mod Bar-Alb (*λ* = 2, *p* = *A* = 0)	0.618	2.55	10.1	36.3	1.96
Var Bar-Alb (*α* = 2, *a* = 0)	1.35	4.46	16.4	−−	−−
Dupl-Div (*p* = 1, *q* = 0.4)	0.349	0.862	2.04	5.01	1.28
Dupl-Div (*p* = 1, *q* = 0.6)	0.155	0.319	0.635	1.31	1.02
Mod Dupl-Div (*p* = 1, *q* = 0.4)	0.340	0.891	2.45	7.09	1.46
Mod Dupl-Div (*p* = 1, *q* = 0.6)	0.165	0.338	0.699	1.44	1.04
Solé (*δ* = 0.25, *α* = 0.005)	4.84	20.5	91.0	436.0	2.16
Solé (*δ* = 0.75, *α* = 0.005)	6.10	20.0	79.5	323.2	1.92
iSite (*p* = 0.5, *q* = 0.01, *r* = 0.8, *I* = 1)	0.114	0.234	0.454	0.925	1.00
iSite (*p* = 0.5, *q* = 0.01, *r* = 0.8, *I* = 2)	0.110	0.216	0.458	0.878	1.01
iSite (*p* = 0.5, *q* = 0.01, *r* = 0.8, *I* = 3)	0.106	0.217	0.432	0.857	1.00
iSite (*p* = 0.5, *q* = 0.01, *r* = 0.8, *I* = 4)	0.107	0.231	0.422	0.848	0.98
iSite (*p* = 0.25, *q* = 0.01, *r* = 0.8, *I* = 4)	0.104	0.249	0.415	0.844	0.98
iSite (*p* = 0.75, *q* = 0.01, *r* = 0.8, *I* = 4)	0.108	0.216	0.437	0.867	1.00
Mod iSite (*p* = 0.5, *q* = 0.1, *r* = 0.8, *s* = 0.1, *I* = 4)	0.288	0.560	1.102	2.53	1.04

The time complexity of the remaining algorithms is *O*(*n*), and this is found consistently, except for the Duplication-Divergence algorithm for *q* = 1 and *q* = 0.4 (and also for the modified implementation of this algorithm). In these cases the algorithm samples denser networks (see Fig 7) which takes up larger amounts of memory, making the implementation less efficient.

**Fig 16:**
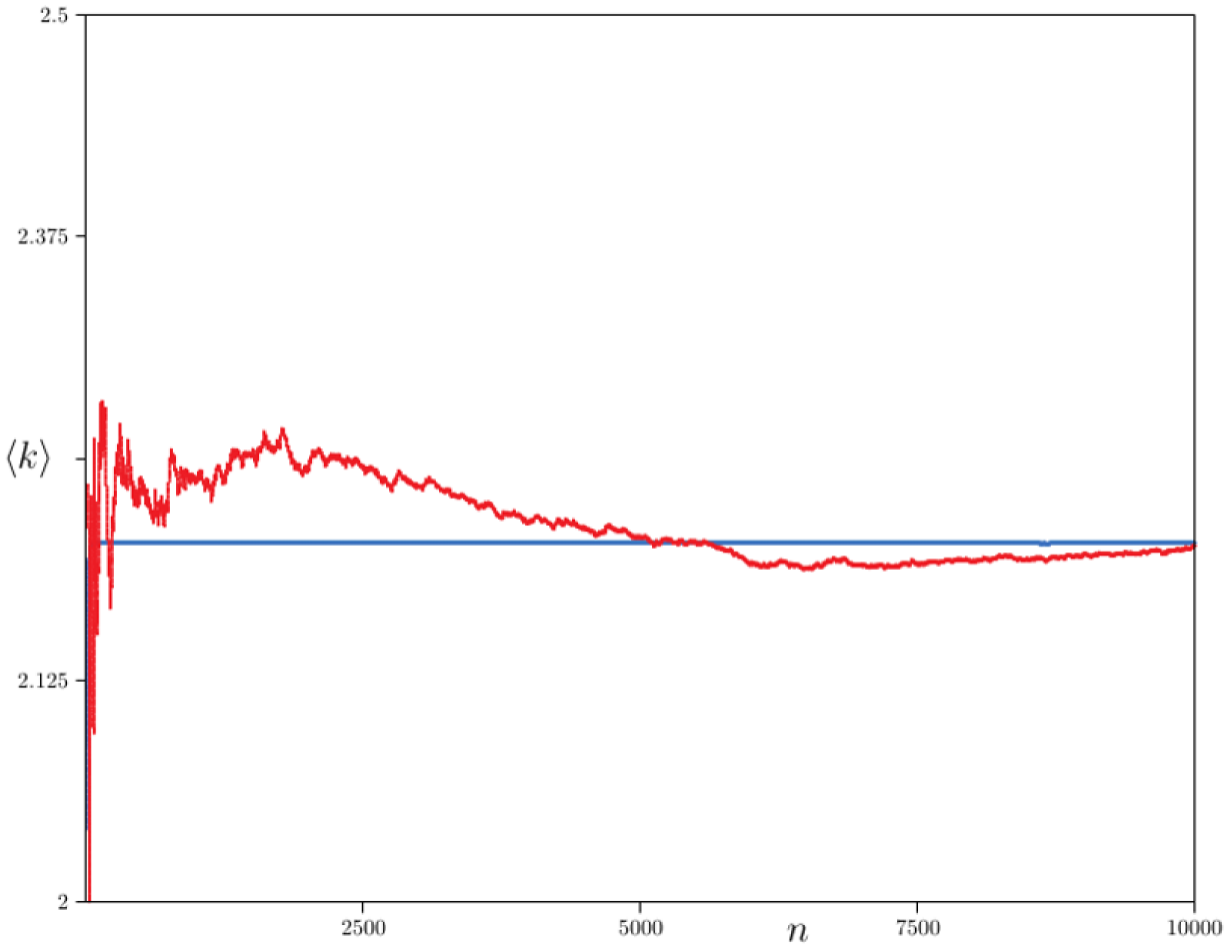
Self-averaging of the connectivity of Barabási-Albert networks. The connectivity of a single network grown with the Barabási-Albert algorithm with *p* = 0.6 as a function of the size of the network is given by the noisy red curve as the network is grown to order *n* = 10000. The blue curve is the average connectivity of Barabási-Albert networks, plotted as a function of *n*. Notice that the red data appear to converge, with increasing *n* to the average, so that the connectivity of a randomly grown Barabási-Albert network appears to converge to its average.

The results in this paper raise some questions about the sampling of scale-free networks by random iterative growth algorithms:

- In some cases, see for example reference [11], the parameters of the algorithms were set to grow networks with properties similar to that of real protein interaction networks. The values of the parameters are then used to estimate the rate of subfunctionalization (or mutation) in the genome. The results are dependent on the algorithm, and so further refinement of algorithms may be needed before useful estimates can be made.
- The mean field approaches are useful in some models (for example the Barabási-Albert algorithm, and the iSite algorithm), but are poorer approximations in other models (the variant Barabási-Albert algorithm, the Duplication-Divergence algorithm and its modification, and the Solé algorithm). Can the mean field approach be improved to give a better approximation to these algorithms?
- Investigation of some numerical properties of the networks (for example the connectivity) suggests that the algorithms may be self-averaging. That is, networks are generated with properties which converge to the statistical averages of these properties over a sample of networks generated by the algorithm. This is, for example, illustrated in Fig 16 for the connectivity of Barabási-Albert networks. As the network is grown, its connectivity appears to approach the average connectivity over a large sample of networks.
- In this paper some algorithms were modified in ways not done before in the literature (this includes the modified Barabási-Albert, the Duplication-Divergence, the Solé and iSite models). Exploring the properties of these modified algorithms, including their usefulness as models of networks in molecular biology, will be the subject of future investigation.

Lastly, these algorithms grow networks using a probabilistic set of rules to implement an elementary move. Each realised network *N*_*n*_ of order *n* is obtained with some probability *p*(*N*_*n*_), so that the function *p*(*N*_*n*_) is a probability distribution over networks of order *n*. Determining *p*(*N*_*n*_) for any of the algorithms presented here seems difficult, and general properties of *p*(*N*_*n*_) remain unknown (other than averages of network properties over *p*(*N*_*n*_) are scale-free if the algorithm grows scale-free networks).
